# Highly active oxygen reduction non-platinum group metal electrocatalyst without direct metal–nitrogen coordination

**DOI:** 10.1038/ncomms8343

**Published:** 2015-06-10

**Authors:** Kara Strickland, Elise Miner, Qingying Jia, Urszula Tylus, Nagappan Ramaswamy, Wentao Liang, Moulay-Tahar Sougrati, Frédéric Jaouen, Sanjeev Mukerjee

**Affiliations:** 1 Northeastern University Center for Renewable Energy Technology, Department of Chemistry and Chemical Biology, Northeastern University, 317 Egan Research Center, 360 Huntington Avenue, Boston, Massachusetts 02115, USA; 2 Department of Biology, Northeastern University, 134 Mugar Life Sciences, 360 Huntington Avenue, Boston, Massachusetts 02115, USA; 3 Institut Charles Gerhardt de Montpellier—UMR 5253, 2 Place Eugéne Batallion, Montpellier Cedex 5 34095, France

## Abstract

Replacement of noble metals in catalysts for cathodic oxygen reduction reaction with transition metals mostly create active sites based on a composite of nitrogen-coordinated transition metal in close concert with non-nitrogen-coordinated carbon-embedded metal atom clusters. Here we report a non-platinum group metal electrocatalyst with an active site devoid of any direct nitrogen coordination to iron that outperforms the benchmark platinum-based catalyst in alkaline media and is comparable to its best contemporaries in acidic media. *In situ* X-ray absorption spectroscopy in conjunction with *ex situ* microscopy clearly shows nitrided carbon fibres with embedded iron particles that are not directly involved in the oxygen reduction pathway. Instead, the reaction occurs primarily on the carbon–nitrogen structure in the outer skin of the nitrided carbon fibres. Implications include the potential of creating greater active site density and the potential elimination of any Fenton-type process involving exposed iron ions culminating in peroxide initiated free-radical formation.

## Abstract (web summary)

The active site of many non-noble metal cathodic oxygen reduction catalysts consists of a nitrogen-corodinated transition metal. Here, the authors report an iron-based electrocatalyst devoid of iron–nitrogen coordination, and demonstrate its high activity in acid and alkaline media.

## Graphical abstract

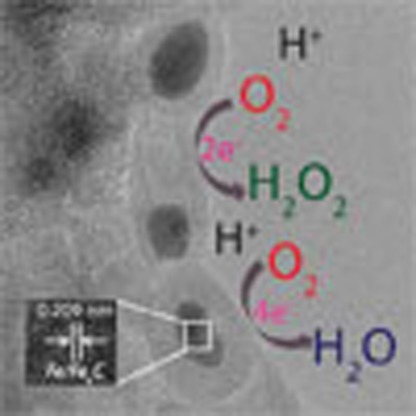

Replacement of noble metal electrocatalysts such as those used in oxygen reduction reaction (ORR) is a research goal for electrochemists involved in the low and medium temperature proton-conducting membrane fuel cells. The high efficiency and theoretical energy density of the H_2_/air fuel cell technology makes it a potential alternative for the replacement of internal combustion engine-based power sources in applications such as transportation and decentralized power and so on.

Three decades of effort towards engendering non-platinum group metal (non-PGM) electrocatalyst has seen a slow but promising evolution of activity for ORR as well as our general understanding of the catalytic active site. Most of these prior efforts have relied on the presence of nitrogen-coordinated sites with or without a transition metal, such as Fe. Despite making progress the current state of the art non-PGM electrocatalyst has yet to achieve the catalytic activity of platinum and thus remains an important research area.

Among the key breakthroughs in this arena were those by Jasinski *et al*.^1^, who was the first to report the oxygen reduction capabilities of cobalt phthalocyanine in basic media. This was followed with that of Jahnke *et al*.^2^, who reviewed the activity of various N_4_-metal chelates and recognized the necessity of heat treatments that resulted in the improvement of stability in basic media and significant enhancement of activity and stability in acidic media. Heat treatment of an existing metal–nitrogen (M–N_*x*_)-coordinated macrocycles dominated the early research of non-PGMs but Yeager and co-workers^3^ caused a principle shift resulting in a significant jump in activity by transitioning to non-macrocyclic precursor materials. More recently, Zelenay *et al*.^4^,^5^ and Dodelet *et al*.^6^,^7^ have demonstrated significant enhancement using the non-macrocyclic precursor-based active-site evolution route. Generally accepted notion of active sites for ORR, formed as a result of a series of heat treatment and etching steps, involved some form of M–N_*x*_ sites^8^,^9^. In addition, presence of some type of transition metal/metal oxides (those involving some degree of metal–metal bonds) are also typically reported especially for materials heat treated at temperatures >800 °C (ref. 10). It was generally believed that these moieties were catalytically inactive due to their thermodynamic instability in low pH environment or their inaccessibility to oxygen if isolated/protected from the electrochemical environment by layers of carbon^11^,^12^. More recently, their direct involvement in acidic media is reported in a cascade of 2e^−^ reduction steps via peroxide intermediate^13^,^14^,^15^.

The role of transition metal/metal oxides has, however, been debated, for example, Kramm *et al*.^16^ claimed that the formation of metal/metal oxides was detrimental to the overall activity of their non-PGM electrocatalyst. Using Mössbauer spectroscopy, they investigated Fe-based non-PGMs and identified five different types of iron species present, but attributed activity to only two types of the iron, both of which were coordinated to nitrogen.

While most of the recent non-PGM literature has focused on a metal-based active sites, there have been several reports of nitrogen-doped (N-doped) carbons with ‘metal-free' active sites^17^,^18^. Stevenson *et al*.^19^ elucidated the ORR pathway in neutral and basic media and found nitrogen-doping-enhanced ORR activity due to improved adsorption of O_2_ and decomposition of peroxide intermediate. Ozkan *et al*.^20^ were the first to demonstrate that N-doped carbons have ORR activity in acidic media and attributed this activity to the increased edge planes of the graphite with pyridinic nitrogen. Dai *et al*.^21^ investigated a N-doped graphene in alkaline media and provided further proof of these ‘metal-free' active sites. Deng *et al*.^22^ reported a material consisting of iron nanoparticles (Fe NPs) encapsulated in carbon nanotubes (CNTs) that was shown to effectively catalyse ORR in the acidic environment of a proton exchange membrane (PEM) fuel cell. Their density function theory (DFT) calculations revealed that the interaction between the Fe and the C, in the wall of the CNT, significantly enhanced the activity of the CNTs for ORR catalysis. These examples constitute a small sample from the literature of a growing consensus that there are ‘metal-free' active sites present in non-PGMs. However, the synthesis procedure for all of previously reported electrocatalysts invariably involve a metal component (typically iron) and the final electrocatalyst cannot be declared completely devoid of Fe NPs and/or iron carbides (Fe_3_C)^4^,^23^,^24^. Since these moieties are present in the electrocatalyst, their role in ORR cannot be ruled out without *in situ* evidence.

This work via our spectroscopic evidence and the use of metal organic framework (MOF) as a template to form active sites with and without transition elements provides evidence of creation of metal-free active sites for ORR with no involvement of active Fe–N_*x*_ sites. In this work, a MOF based on Zn (ZIF-8) is used as an encapsulator for Fe chelated with a nitrogen complex. Encapsulation of the chelated iron complex in the pores is employed in an effort to obtain equal distribution and high density of active sites. ORR is investigated in alkaline and acidic media to better understand the reduction mechanism of the studied electrocatalyst. MOF-based structures with and without incorporation of iron precursor are heat treated and etched to form the active electrocatalyst, enabling proper comparison with samples derived wherein no iron was chelated. This is in contrast to previous reports where a complete absence of iron cannot be claimed^19^,^20^,^21^,^22^,^25^. By combining electrochemical and spectroscopic methods, we are able to show that while the derived iron containing non-PGM catalysts exhibits high ORR activities, coordination environment for iron is predominantly Fe/Fe_*x*_C particulate in nature. Using *in situ* X-ray absorption spectroscopy (XAS), we are able to characterize the iron coordination of the electrocatalyst under actual simulated operating conditions of a PEM fuel cell and are unable to detect any direct Fe–N_*x*_-coordinated sites present in the non-PGM electrocatalyst. In addition to the absence of direct Fe–N_*x*_ coordination, we also show the complete absence of any direct involvement of the Fe in the electrocatalytic pathway as a result of its presence subsurface to the carbo-nitrided fibres and hence its important implication in avoidance of any Fenton-type process related to onset of peroxide-induced free-radical formation as detailed earlier^26^.

## Results

### Physicochemical characterization

The 2*θ* values and symmetry of the powder XRD of FePhen@MOF (before heat treatment) as compared with the purchased Basolite (Sigma, Basolite Z1200) confirm the formation of the crystalline MOF structure (Supplementary Fig. 1). The concomitant diminished intensity of the signal from FePhen@MOF (Supplementary Fig. 2) is indicative of the presence of guest molecules within the MOF structure as a result of encapsulation (Supplementary Note 1), this is in agreement with similar observations reported earlier^27^. In addition, the absence of the diffraction lines characteristic of 1,10-phenanthroline (Supplementary Fig. 1; N-chelating agent for Fe) imply that it is not present as a separate entity in crystalline form. However, ultraviolet–visible (ultraviolet–vis) spectroscopy (Supplementary Table 1) confirmed retention of 1,10-phenanthroline in the FePhen@MOF and thermogravimetric analysis indicated its enhanced thermal stability as a result of the encapsulation (Supplementary Fig. 3). Further, FePhen@MOF exhibited a much smaller Brunauer–Emmet–Teller (BET) (Supplementary Fig. 4) surface area than the commercial analogous Basolite (424 versus 1813, m^2^ g^−1^), which is likely attributable to the encapsulation of the chelated iron complex within the MOF pore reducing the overall pore volume (Supplementary Fig. 5).

**Figure 1:**
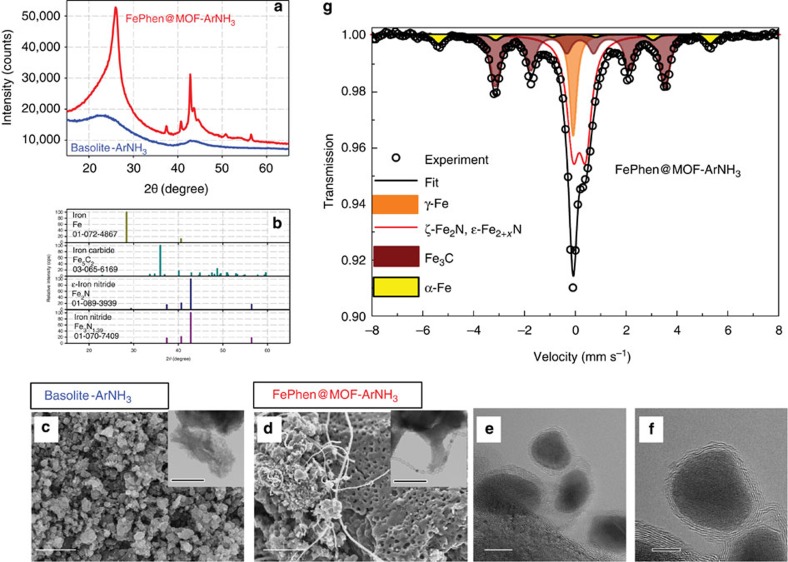
Physicochemical characterization. (**a**) X-ray diffraction pattern of FePhen@MOF-ArNH_3_ and Basolite-ArNH_3_. (**b**) Diffraction patterns of iron, iron carbide and iron nitrides. (**c**) SEM image of Basolite-ArNH_3_. Scale bar, 2 μm. Inset TEM image of amorphous carbon. Scale bar, 100 nm. (**d**) SEM image of FePhen@MOF-ArNH_3_. Scale bar, 2 μm. Inset TEM image of bamboo-jointed CNT. Scale bar, 100 nm. (**e**) HR-TEM image of iron encapsulated in bamboo joints of CNT in FePhen@MOF-ArNH_3_. Scale bar, 10 nm. (**f**) HR-TEM image of Fe/Fe_*x*_C nanoparticle surrounded by graphite layers. Scale bar, 5 nm. (**g**) ^57^Fe Mössbauer spectrum of FePhen@MOF-ArNH_3_ measured at room temperature.

The FePhen@MOF material was subjected to an initial heat treatment (1,050 °C) in Ar atmosphere (FePhen@MOF-Ar) for a tailored breakdown of the MOF structure and removal of Zn nodes via sublimation resulting in an initial rendering of a carbonaceous material with significant electronic conductivity. The XRD powder pattern (Fig. 1a; Supplementary Fig. 6) shows emergence of an elevated baseline typical for presence of amorphous material as well as diffraction lines of graphite and some iron phases (Fig. 1b; iron-Fe (Im-3 m) and iron carbide-Fe_5_C_2_ (C2/c)). However, we were not able to accurately determine the exact stoichiometry of the iron carbide using XRD. An additional heat treatment (1,050 °C) in ammonia (FePhen@MOF-ArNH_3_) led to the formation of several new diffraction lines (Supplementary Fig. 6) that indicate the incorporation of nitrogen into the carbon scaffold in the vicinity of the iron. These new diffraction lines post NH_3_ heat treatment show emergence of Fe nitrides (Fe_3_N_1.59_ (P6322) and Fe_2_N (P-31 m(162))); however, XRD alone could not unambiguously determine the exact nitrogen content. Comparing these XRD results with those obtained from Basolite-ArNH_3_ (subjected to the same heat treatments as FePhen@MOF-ArNH_3_; Fig. 1a) lead us to believe that in the latter case the iron is converted to its metallic form during the initial argon heat treatment and subsequently acts as a catalyst that graphitizes the vicinal carbon–nitrogen's from MOF precursor. The second heat treatment in ammonia, thus, is effective in etching a portion of the remaining amorphous carbon^28^ and introducing nitrogen functionalities into the carbon scaffold. The BET surface area increased with each heat treatment (Supplementary Fig. 4) resulting in FePhen@MOF-ArNH_3_ exhibiting a surface area of 1,200 m^2^ g^−1^, comparable to that of the original Basolite (no heat treatment). Basolite and FePhen@MOF are originally microporous (<20 Å) materials; however, the microporosity is increased with each heat treatment and mesopores (20–40 Å) are introduced (Supplementary Fig. 7) to FePhen@MOF-ArNH_3_.

XAS was used to further probe the local coordination of the iron present after heat treatment (Supplementary Note 2). Fourier transform (FT) of the extended EXAFS of the Fe K-edge measured on the electrode *in situ* (held at 0.3 V, free of adsorbates) was dominated by a peak at ∼2.2 Å, which can be fit with Fe–Fe scattering (Supplementary Fig. 8; Supplementary Table 2). Qualitative comparison of the FePhen@MOF-ArNH_3_ spectrum with the spectra of the standard metallic iron foil (Fe foil) and a Fe_3_C standard suggests that the iron is predominantly converted to its metallic state with a minor carbide phase. However, it should be noted that an inherent limitation of XAS is the inability to differentiate between C, N and O neighbouring atoms (Supplementary Note 3). Therefore, we cannot use FT EXAFS to conclusively identify the presence or absence of Fe nitrides or carbides in the final catalyst.

The different iron coordination environments in FePhen@MOF-ArNH_3_ were further investigated with *ex situ*^57^Fe Mössbauer spectroscopy (Fig. 1g). The absorption spectrum measured at room temperature (RT) was successfully fit with four components: (1) paramagnetic γ-Fe, (2) ferromagnetic Fe_3_C, (3) ferromagnetic α-Fe and (4) ξ-Fe_2_N. (Supplementary Table 3; Supplementary Note 4).

The surface morphology of FePhen@MOF-ArNH_3_ (Fig. 1d) was probed with SEM and revealed that the heat treatment produces several different carbon morphologies. The decomposition of the MOF framework and evaporation of Zn above 550 °C (ref. 29) forms a porous carbon framework. In addition, some of the Fe agglomerated into Fe NPs that catalysed the growth of CNTs of varying sizes and possessed distinct bamboo-like joints, which are characteristic of N-doped carbon^19^,^30^. High-resolution TEM revealed that Fe-particles were contained within the compartments of the CNTs (Fig. 1e,f) and the compartment wall thickness varied with the size of the Fe-particles. Conversely, the surface morphology of Basolite-ArNH_3_ (Fig. 1c) had no CNTs present, which suggests that the iron present in FePhen@MOF primarily acts as a catalyst for the graphitization of carbon.

### Electrochemical performance

**Figure 2:**
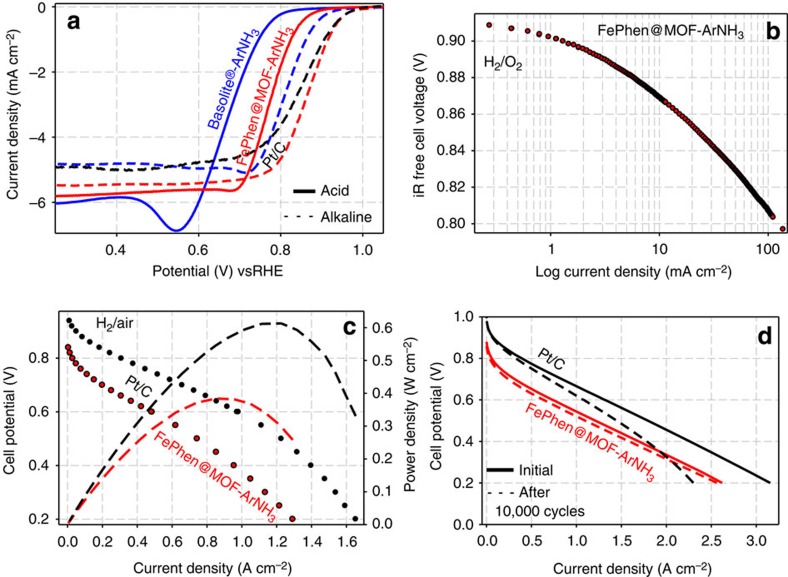
RDE and fuel cell testing. (**a**) Rotating disk electrode (RDE) ORR polarization plots collected with FePhen@MOF-ArNH_3_, Basolite-ArNH_3_ and Tanaka Pt/C in O_2_ saturated electrolyte (0.1 M HClO_4_ and 0.1 M KOH) at 20 mV s^−1^ with rotation rate of 1,600 r.p.m. at room temperature. (**b**) H_2_/O_2_ fuel cell iR-corrected Tafel plot for FePhen@MOF-ArNH_3_ cathode (loading of 3 mg cm^−2^) using Nafion (NRE 211) membrane. (**c**) H_2_/air fuel cell polarization curve and corresponding power density curve for same MEA from **a**. A Pt/C (0.4 mg_Pt_ cm^−2^ loading) cathode reference was used for comparison. (**d**) H_2_/O_2_ fuel cell polarization curves of FePhen@MOF-ArNH_3_ and Pt/C cathode measured initially and after 10K cycles (using 3 s hold and pulses between 0.6 and 1.0 V under nitrogen flow).

Basolite (Basolite-ArNH_3_) was subjected to the same heat treatment as FePhen@MOF-ArNH_3_ (that is, Ar followed with NH_3_ at 1,050 and 1,050 °C, respectively) to be used as a reference for a Fe-free catalyst in our rotating ring-disk electrode (RRDE) studies. Typically, Pt/C is known to proceed predominantly through the 4e^−^ path in both alkaline and acidic media, as long as the surface is free of poisonous adsorbates^31^. However, in alkaline media, it has been shown that specifically adsorbed hydroxyl species non-covalently interact with solvated oxygen molecules and the reduction proceeds through a 2e^−^ outer sphere charge transfer process producing the peroxide anion intermediate (HO_2_^−^), (as detected at the ring electrode of RRDE at ∼0.8 V (ref. 32); Supplementary Fig. 9) followed with an additional concerted 2e^−^ reduction step to water. The same report showed that in contrast to this a non-PGM catalyst derived from heat-treated Fe-tetraphenylporphyrin (Fe-TPP)^32^ exhibits an exclusive inner sphere concerted 4e^−^ reduction in alkaline media due to direct adsorption of O_2_ and the kinetically favoured hydrogen peroxide reduction reaction^32^. Central to this mechanism was its correlation with Fe–N_*x*_ (predominant four coordination) active sites formed in the divacant defects in the carbon basal plane^32^. In alkaline media, Basolite-ArNH_3_ has an onset potential of 0.97 V (Fig. 2a) and the oxidation of the peroxide intermediate detected at the ring electrode coincides with the onset of reduction at the disk (Supplementary Fig. 9). This indicates that the peroxide is not stabilized on the active site and ORR proceeds predominantly through a 2e^−^ reduction pathway. However, the presence of the iron in FePhen@MOF-ArNH_3_ provides a 60 mV improvement (with respect to Basolite-ArNH_3_) in the onset potential (1.03 versus 0.97 V) as well as a 40 mV improvement in half-wave potential (*E*_½_) (0.86 versus 0.82 V; Fig. 2a). In addition, the onset of the peroxide oxidation peak at the ring electrode is shifted cathodically to ∼0.45 V (Supplementary Fig. 9). Peroxide oxidation at 0.45 V is also observed on Pt/C electrocatalyst (Supplementary Fig. 9) and has been attributed to 2e^−^ reduction of oxygen by the quinone functionalities of the carbon support^32^. However, FePhen@MOF-ArNH_3_ did not demonstrate enhanced hydrogen peroxide reduction reaction activity (Supplementary Fig. 10) and therefore this increase in activity cannot be attributed to peroxide specific Fe–N_*x*_ sites. These electrochemical results suggest incorporation of iron moieties in FePhen@MOF-ArNH_3_ in a different coordination environment as compared with classical Fe–N_*x*_ active-site-based electrocatalyst (as exemplified by Fe-TPP). Considering the XRD, SEM and TEM results presented above, it can be conjectured that Fe is present in the form Fe/Fe_*x*_C, subsurface to the N-doped carbon overlayer. As shown later in this article based on *in situ* element-specific XAS spectroscopy results, subsurface Fe/Fe_*x*_C are inactive towards O_2_ adsorption, and the ORR activity predominantly appears to arise from the N-doped carbon surface. It appears that the presence of the subsurface Fe/Fe_*x*_C acts to stabilize the peroxide intermediate on the active site in the alkaline medium.

When the same experiment was conducted in acidic media, FePhen@MOF-ArNH_3_ exhibited a 90 mV improvement in *E*_½_ (0.77 versus 0.68 V) for ORR when compared with Basolite-ArNH_3_ (Fig. 2a). This is evidenced by the higher onset potential (*E*=0.93 versus *E*=0.89 V) of FePhen@MOF-ArNH_3_ versus Basolite-ArNH_3_ in oxygen saturated 0.1 M perchloric acid (HClO_4_). More interestingly, the onset of peroxide oxidation for both of the non-PGM electrocatalyst in acidic media now coincides with the onset of ORR (Supplementary Fig. 11). When switching to the low pH environment, the peroxide intermediate is no longer present as anion, but as a neutral species (H_2_O_2_) that has been shown to desorb more easily into the bulk electrolyte due to the absence of intermediate stabilization on the active site^32^. Our results confirm this finding, but we also find that the oxidative ring current of Basolite-ArNH_3_ is an order of magnitude higher than FePhen@MOF-ArNH_3_. This suggests that the presence of the iron moieties in FePhen@MOF-ArNH_3_ still promotes the selectivity towards the 4e^−^ ORR pathway in acidic media. As mentioned above in our discussions on alkaline media results, subsurface Fe appears to be critical in the electrocatalysis in close concert with synergistic effect of N-doped carbon (Fig. 1e,f). Independent DFT calculations^22^,^33^ conducted on distinct Fe-based non-PGM catalysts support our theory that the electronic properties of the carbon could be enhanced by the interaction with nitrogen and Fe enabling easier adsorption of O_2_ and reduction to H_2_O.

The inherent ORR activity exhibited by RRDE measurements of FePhen@MOF-ArNH_3_ in acidic media (Fig. 2a) are corroborated by single PEM fuel cell measurements. Polarization and durability curves were collected, on a membrane electrode assembly (MEA) employing a FePhen@MOF-ArNH_3_ cathode, under the US Department of Energy (US DOE) suggested testing conditions of 80 °C, 100% relative humidity, 1 bar partial pressure of H_2_/O_2_ (1.5 bar total pressure) and/or 0.4 bar partial pressure of O_2_ in H_2_/air (2.5 bar total pressure). In a H_2_/O_2_ fuel cell, FePhenMOF-ArNH_3_ MEA generated a kinetic current density of >100 mA cm^−2^ at 0.8 V_iR free_ (Fig. 2b), ranking it as one of the most active non-PGM cathodes reported to date with no FeN_*x*_ moieties (as demonstrated in the following section). Non-PGM cathodes require higher catalyst loadings (3 mg cm^−2^ compared with 0.4 mg_Pt_ cm^−2^ for Pt/C) that traditionally introduces mass-transport limitations; however, FePhen@MOF-ArNH_3_ was able to reach 75% the power density of a state-of-the-art Pt/C cathode run under the same conditions. Although microporosity is considered a prerequisite for good catalytic activity in non-PGMs, mesopores are essential for effective mass transport, and we believe the increase in mesoporosity (Supplementary Fig. 7) on heat treatment imparted superior mass-transport properties that result in the enhanced activity. On switching to air as the oxidant the FePhen@MOF-ArNH_3_ MEA (Fig. 2c) reached a kinetic current density of 50 mA cm^−2^ at 0.8 V_non-iR corrected_ and a peak power density of 0.38 W cm^−2^ at 0.4 V (or a current density of 1 A cm^−2^, the current DOE performance target), ∼60% the power density reached by the Pt-based cathode under the same operating conditions. To our knowledge, this is the highest reported H_2_/air activity to date by a non-PGM cathode containing no direct Fe–N_*x*_ coordination (as detailed in subsequent sections). In addition to the exceptional performance, the most impressive characteristic of FePhen@MOF-ArNH_3_ was the outstanding stability observed through 10,000 potential cycles (Fig. 2d; protocol in accordance to method shown in Fig. 2 and described in Methods section) that simulates automotive drive cycle conditions. When compared with the state-of-the-art Pt/C MEA (Fig. 2d) subjected to the same test, FePhen@MOF-ArNH_3_ MEA proved to be more durable (100 mA cm^−2^ versus 250 mA cm^−2^ (for Pt/C reference) current loss at 0.6 V_non-iR corrected_) and maintained its superior mass-transport properties. Increased carbon graphitization has been reported to improve resistance to carbon corrosion^34^ and one source of enhanced stability of FePhen@MOF-ArNH_3_ could be attributed to the presence of CNTs that were observed by SEM/TEM. A variety of harsh testing conditions, such as cycling in the presence of hydrogen peroxide (Supplementary Note 6), have substantiated the findings mentioned above, however only a few representative examples are shown in the present work. The exact origin of durability in FePhen@MOF-ArNH_3_ requires more extensive investigation and will be presented in a future publication.

### Identification of Fe-coordination

**Figure 3:**
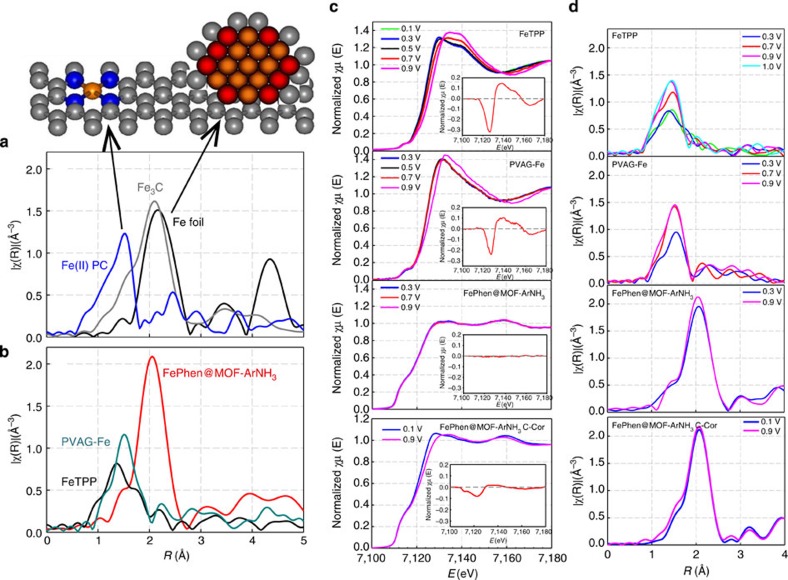
Structural and electronic investigation of iron in non-PGMs. (**a**) Fourier transform EXAFS of Fe(II)PC, Fe_3_C and Fe foil standards collected at Fe K-edge (7,112 eV) (**b**) Fourier transform EXAFS of FePhen@MOF-ArNH_3_, FeTPP pyrolyzed at 800 °C and PVAG-Fe in N_2_ saturated 0.1 M HClO_4_. (**c**) Potential-dependent normalized Fe K-edge XANES of FeTPP, PVAG-Fe, FePhen@MOF-ArNH_3_ and carbon-corroded Fe-Phen@MOF-ArNH_3_ collected in N_2_ saturated 0.1 M HClO_4_; inset difference spectra, Δ*μ*=*μ* (0.9 V)−*μ* (0.3 V). (**d**) Potential-dependent FT-EXAFS of same electrocatalysts in **b** collected in O_2_ saturated 0.1 M HClO_4_.

Earlier observations of heat-treated Fe-TPP^32^ (heat treatment of existing macrocycle) and PVAG-Fe^14^ (Fe-based catalysts prepared from precursors using a reactive polymer approach) clearly indicated the presence of Fe–N_*x*_ active sites in addition to relatively small but stable Fe NPs in both the electrocatalysts despite the different precursors/synthetic routes used. They also represent different types of defect structures on the carbon and hence Fe spin states. Among the key features associated with ORR on such Fe–N_*x*_ containing electrocatalyst elucidated using *in situ* XAS are (a) the Fe^2+^–N_4_ active site undergoes a redox transition to (H)O-Fe^3+^-N_4_ between 0.7 and 0.9 V versus RHE, which correlates with an edge shift of the XANES characteristic of such a change in oxidation state with increasing potential (Fig. 3c,d), and (b) a concomitant increase in the magnitude of the FT peak at ∼1.6 Å (associated with Fe–N/O scattering) in the same corresponding potential showing oxide formation on the Fe in oxidation state 3 (Fig. 3d). Fe^III^–N_*x*_ (OH) formation close to 0.9 V versus RHE clearly indicated by the forward FT at Fe K-edge (Fig. 3d) also means that the onset of oxygen reduction is intimately related to such a redox transition (this is also supported by redox peaks in alkaline and acidic media). In contrast to these observations on non-PGM catalysts containing Fe–N_*x*_ coordination, the Fe K-edge FT of the EXAFS for FePhen@MOF-ArNH_3_ does not contain the characteristic Fe–N/O peak at 1.6 Å (indicative of Fe–N interaction at and below 0.7 V and an additional Fe–O(H) interaction at higher potentials) and is characterized instead by a peak at ∼2.2 Å coinciding with Fe–Fe scattering as seen by comparison with characteristic corresponding peaks from metallic Fe and Fe_3_C standards (Fig. 3a). Furthermore, when the applied potential is increased, the Fe K-edge XANES energy of FePhen@MOF-ArNH_3_ remains unchanged. XANES is far more sensitive than EXAFS at detecting the presence of FeN_*x*_ species (Supplementary Note 3) and our specialized *in situ* technique further enhances this response. Moreover, analysis of the ^57^Fe Mössbauer spectroscopy results unambiguously confirm the absence of FeN_*x*_ moieties in FePhen@MOF-ArNH_3_. Doublets D1 and D2, identified in all Fe-based non-PGM electrocatalysts investigated with Mössbauer spectroscopy to date, were assigned to FeN_*x*_C_y_ moieties covalently integrated in graphene sheets^16^,^35^. However, the doublet observed with FePhen@MOF-ArNH_3_, with isomer shift (IS) and quadrupole splitting values of 0.16 and 0.50 mm s^−1^, respectively, is fundamentally different and can be confidently and entirely assigned to a nitrogen-rich iron nitride crystalline structure (Supplementary Note 5). Hence, it is concluded that FeN_*x*_ moieties integrated in graphene sheets are absent in FePhen@MOF-ArNH_3_. These findings further confirm the results presented above using XRD, SEM and TEM in conjunction with electrochemistry. We believe that FePhen@MOF-ArNH_3_ is predominantly made up of subsurface Fe/Fe_*x*_C nanoparticles and there are no detectable Fe–N_*x*_ moieties present when studied under *ex situ* and *in situ* conditions. The unique characteristics of XAS enable probing of the coordination environment with element specificity (in this case Fe) and *in situ* capability provides incontrovertible evidence of a highly active non-PGM in acidic media that lacks the direct Fe–N_*x*_-coordinated active site. To the author's best knowledge, this represents a unique type of active site where lack of direct metal–nitrogen coordination is unambiguously shown using *in situ*-specific probe of the actual transition metal coordination environment in conjunction with one of the highest reported activity for ORR in acidic and alkaline media.

This conclusion was further supported by subjecting FePhen@MOF-ArNH_3_ electrocatalyst to carbon corrosion cycling in the potential range of 1.0–1.5 V (100 mV s^−1^, acidic media; Supplementary Fig. 12). The carbon-corroded FePhen@MOF-ArNH_3_ has the emergence of the signature edge shift in the Fe K-edge XANES spectra seen previously for FeN_*x*_-coordinated active sites (Fig. 3c) associated with a Fe^II^ to Fe^III^ redox transition (0.7–0.9 V). This clearly indicates the formation of some FeN_*x*_ moieties and their direct involvement with the ORR mechanism. On the basis of these results, we conclude that some of the Fe/Fe_*x*_C nanoparticles covered by fewer graphitic sheets are exposed to the acidic environment during the carbon corrosion treatment and are dissolved. This process would then allow some of the dissolved Fe-ions to either (a) adsorb on nitrogen sites that are doped into the carbon matrix to form the FeN_*x*_ active site, similar to the active-site formation previously proposed by Yeager *et al*.^31^,^36^,^37^, or (b) form Fe^III^ hydroxides (Fe(OH)_3_) as proposed by Goellner *et al*.^38^

These results also clearly point towards the fact that since FePhen@MOF-ArNH_3_ constitutes of subsurface Fe/Fe_*x*_C-type moieties and their role in any Fenton's-type peroxide-induced free-radical formation is unlikely. Some of the durability results presented (Fig. 2d; Supplementary Figs 13 and 14) are in concert with this observation, especially when compared with Pt/C. This is a very critical distinction from the Fe–N_*x*_-based non-PGM electrocatalysts.

## Discussion

In this work, we introduced a Fe-based non-PGM ORR electrocatalysts, FePhen@MOF-ArNH_3_, which utilizes a MOF support that acts as a host for the Fe-based sites. Orderly dispersion of the Fe-chelates is achieved with the encapsulation synthesis and gives rise to an electrocatalyst with high activity for ORR in acidic media and outperforms the benchmark Pt/C catalyst in alkaline media. The exceptional ORR activity measured using a RRDE setup in acid translated to the superior PEM fuel cell performance where FePhen@MOF-ArNH_3_ showed excellent mass-transport properties and stability. Characterization techniques revealed the iron is present as Fe/Fe_*x*_C nanoparticles that are subsurface to N-doped carbon overlayer; however, they could play a role in the electrocatalysis by imparting a synergistic effect on the N-doped carbon that allows stabilization of the peroxide intermediate and enables the full 4e^−^ reduction of oxygen to water. What sets this work apart from others is the definitive proof that this exceptionally active non-PGM electrocatalysts lacks direct Fe–N_*x*_ coordination when studied using *ex situ*^57^Fe Mössbauer spectroscopy and *in situ* synchrotron XAS at the Fe K-edge under simulated conditions of operating PEM cell. All previous reports in the literature have been obtained under *ex situ* conditions alone and cannot account for catalyst structure changes at the catalyst–electrolyte interface with an applied potential. The lack of evidence suggesting the presence of Fe–N_*x*_ moieties under *in situ* conditions allows us to conclusively attribute the majority of the ORR activity to the N-doped carbon structure. On the basis of our results, we believe that the iron first acts as a catalyst for the graphitization of N-doped carbon during the heat treatment in which some iron is integrated into the carbon and enhances the electrocatalytic properties enabling the 4e^−^ reduction of oxygen.

## Methods

### Catalyst synthesis and electrochemical measurements

The commercial Pt/C (46%) electrocatalyst used as a standard in this study is obtained from Tanaka Kikinzoku International KK (Japan). For the synthesis of FePhen@MOF, 2-methylimidazole (5.90 g, 0.072 mol, 160 eq) was dissolved in methanol (20 ml) at RT with stirring in flask A. In flask B, zinc(II) nitrate χ-hydrate (10.80 g, 0.036 mol, 80 eq) and 1,10-phenanthroline monohydrate (12.96 g, 0.072 mol, 160 eq) were dissolved in methanol (30 ml) and water (45 ml) at RT with stirring. Once both flasks' contents were fully dissolved, flask B was added to flask A. Iron(II) acetate (0.081 g, 4.66E−4 mol, 1 eq) was added to the reaction, and this was stirred at RT for 24 h. A second reaction identical to the aforementioned vessel was assembled, and the two separate reactions progressed in tandem. Before the addition of iron(II) acetate, the reaction began to turn slightly milky white/turbid in appearance. This intensified over the course of the reaction, resulting in a fine suspension turbid and pale orange in colour. The two reaction vessels were then combined in 6 × 50-ml centrifuge tubes and centrifuged at 4,000 r.p.m. for 25 min, washed three times with methanol (6−40 ml each time), centrifuging at 3,700 r.p.m. for 17 min in between each washing. The resulting orange/white solid was dried in a vacuum oven for 6–12 h at 60–70 °C and afforded formation of the desired FePhen@MOF, a light tan powder (typically 4.5–5.5 g total yield from the two combined reaction vessels). The dried powder was then subjected to either one or two heat treatments, that is, in argon at 1,050 °C with a 1 h dwell time (FePhen@MOF-Ar) and ammonia at 1,050 °C with an 18 min dwell time (FePhen@MOF-ArNH_3_), respectively. The weight per cent of Fe used in the synthesis of FePhen@MOF and its retention throughout the heat treatment process (FePhen@MOF-Ar and FePhen@MOF-ArNH_3_) was quantified using inductively coupled plasma mass spectrometry (ICP-MS) and is listed in the (Supplementary Table 4). Perchloric acid electrolyte (0.1 M) was prepared using double-distilled 70% perchloric acid (GFS Chemicals) and potassium hydroxide electrolyte (0.1 M) was prepared using pellets (Alfa Aesar).

Electrochemical measurements were carried out on glassy carbon disk (5.61-mm diameter, Pine Instruments) that was polished with 0.05-micron alumina paste (Buehler, Lake Bluff, IL) and then sonicated in distilled water and Isopropyl alcohol. Catalysts inks were prepared by dispersing the catalyst in a volume of 1:1 Millipore water:isopropyl alcohol with 10 vol% of 5 wt% Nafion as a binder. The ink solution was then sonicated ∼60 min to get a uniform suspension. A small volume of the catalyst ink was deposited on the glassy carbon substrate to obtain a platinum metal loading of 15 μg_Pt_ cm^−2^ and a non-PGM loading of ∼600 μg cm^−2^. All electrochemical measurements were carried out at RT (20–25 °C) in a standard electrochemical cell (Chemglass) with an acid or base electrolyte using a rotating disk electrode (RDE) setup from Pine Instrument Company connected to an Autolab bipotentiostat (PGSTAT302N). Cyclic voltammetry was run on both Pt and non-PGM catalysts in 0.1 M HClO_4_ and 0.1 M potassium hydroxide (KOH) bubbled with argon. ORR was investigated by the RDE technique after bubbling oxygen in the electrolyte solution followed with rotations at 100, 400, 900, 1,600 and 2,500 r.p.m. Scans were recorded at 20 mV s^−1^ and all potentials are referenced to a reversible hydrogen electrode (RHE) scale made from the same solution as the electrolyte. Carbon corrosion treatment of FePhen@MOF-ArNH_3_ was performed in argon saturated 0.1 M HClO_4_ by cycling 1–1.5 V versus RHE at 100 mV s^−1^ and 1,600 r.p.m. rotation speed for 7,500 cycles. Hydrogen peroxide cycling test involves cycling (0.05–1.2 V versus RHE) the catalyst under RDE conditions (600 μg cm^−2^), 0.5 M H_2_SO_4_ with and without 70 mM H_2_O_2_.

MEAs for fuel cell testing were prepared using FePhen@MOF-ArNH_3_ cathode on gas diffusion layer (GDL, Sigracet BC25 GDL), Pt/C (Tanaka, 46%) anodes on GDL (Sigracet BC25), Nafion 5 wt% ionomer solution, and Nafion 211 ionomer membrane. To prepare the cathode, a catalyst ink composed of FePhen@MOF-ArNH_3_ dispersed in a water–alcohol mixture with the requisite amount of ionomer (Nafion 5 wt%) was sprayed on GDL (Sigracet, BC25). Typical cathode loading consisted of 0.3 mg cm^−2^ with an ionomer content of 60% by weight of the catalyst. After drying the cathodes, a layer of interfacial ionomer was sprayed to achieve a loading of 0.4 mg_Nafion_ cm^−2^. Anodes were prepared with commercial Pt/C (Tanka, 46%) in the same manner as the cathode. Electrodes for the Pt-based cathode MEA were supplied by industry. Hot pressing of the electrodes together with a Nafion 211 membrane was carried out at 130 °C and 1,000 lbs for duration of 5 min. MEAs were then assembled in a fuel cell consisting of 5 cm^2^ serpentine flow fields. Humidification of the MEA was performed for 60 min by flowing N_2_ (100% relative humidity (RH)) at a cell temperature of 80 °C. The operating temperature of the fuel cell was 80 °C and the cell was activated with H_2_/O_2_ (inlet temperatures 85 °C, 100% RH, 22 p.s.i. back pressure). Durability cycling followed protocols from Nissan Technical Center North America designed to simulate accelerated drive cycle conditions based on transitions between open circuit voltage and idling in automobile operation. Durability was tested at 80 °C with N_2_ flowing on the cathode while stepping potential from 0.6 to 1.0 V versus RHE with 3 s hold at each potential step.

### Physicochemical characterization

X-ray diffraction was performed on a Rigaku (model Ultima-IV) diffractometer with Cu Kα radiation (*λ*=1.5418 Å) at 40 kV and 40 mA. The scan speed was 2–20 s and the step size was 0.1°. ultraviolet–vis quantification performed on Thermo-Fisher ultraviolet–vis spectrophotometer collecting absorbance spectra from 190 to 700 nm. Calibration curve generated from 12.5 to 100.0 μM 1,10-phenanthroline monohydrate dissolved in 1 M HCl. Three hundred micromolar solutions of FePhen@MOF and Basolite Z1200 standard dissolved in 1 M HCl were run under the same conditions. Thermogravimetric analyses were performed on TA Instruments SDT Analyzer Q600 from 22 to 1,100 °C with a ramp rate of 5 °C min^−1^ and dwelling for 5 min at maximum temperature. All studies were conducted under argon atmosphere, with a flow rate of 100 ml min^−1^. N_2_ sorption analysis was performed on a Quantachrome NOVA 2,200e at 77 K. Total surface area was determined by the BET method and pore size distribution was determined using non-local DFT split pore method from the NovaWin software. SEM was performed on a Hitachi S-4,900 FSEM instrument with an accelerating voltage of 3–5 keV with samples mounted on a carbon-adhesive stub attached to an aluminum sample stage. High-resolution TEM images were taken on a JEOL 2010 field emission gun (FEG) TEM at 200 kV with samples deposited on a holey carbon film on a 300 mesh copper grid.

^57^Fe Mössbauer spectroscopy was measured with a ^57^Co source embedded in rhodium matrix. The measurement was performed keeping the source and absorber at RT. The spectrometer was operated with a triangular velocity waveform, and a gas filled proportional counter was used for the detection of the γ-rays. Velocity calibration was performed with an α-Fe foil. The spectra were fitted with a combination of Lorentzian lines. In this way, spectral parameters such as the IS, the electric quadrupole splitting, the linewidth at half maximum, the hyperfine fieldsand the absorption spectral areas of the various spectral components were determined. No constraints were applied to the fitting parameters except on the IS of the singlet assigned to γ-Fe, fixed at −0.08 mm s^−1^ on the basis of previous experimental studies on Fe–N–C catalysts showing the presence of the ubiquitous singlet assigned to γ-Fe.

The *in situ* XAS studies at the Fe K-edge (7,112 eV) were performed at X3B beamline of National Synchrotron Light Source (Brookhaven National Laboratory, NY, USA). A specially designed spectro-electrochemical cell was employed^39^. The Fe K-edge spectra were collected in fluorescence mode with a 32-element Ge solid state detector as a function of potential in the range 1.0–0.1 V. A pseudo steady state was established by holding the cell for ∼5 min before collecting the spectra. The electrolyte, 0.1 M HClO_4_, was saturated with either argon or oxygen. Athena^40^ and Artemis^41^ programs were used to process and fit the data. IFEFFIT suite^40^ was used to calibrate, align and normalize the scans. FEFF6 code^42^ was used to calculate the scattering paths to model the χ(R) transforms.

## Additional information

**How to cite this article**: Strickland, K. *et al*. Highly active oxygen reduction non-platinum group metal electrocatalyst without direct metal–nitrogen coordination. *Nat. Commun.* 6:7343 doi: 10.1038/ncomms8343 (2015).

## Supplementary Material

Supplementary Information Supplementary Figures 1-14, Supplementary Tables 1-4, Supplementary Notes 1-6 and Supplementary References
