# Supplementary material for: Highly active oxygen reduction non-platinum group metal electrocatalyst without direct metal–nitrogen coordination
Source: Nat Commun. 2015 Jun 10;6:7343. doi: 10.1038/ncomms8343 (PMC4490352; doi:10.1038/ncomms8343)
Supplement: Supplementary Information — Supplementary Figures 1-14, Supplementary Tables 1-4, Supplementary Notes 1-6 and Supplementary References [file ncomms8343-s1.pdf]

Supplementary Figures

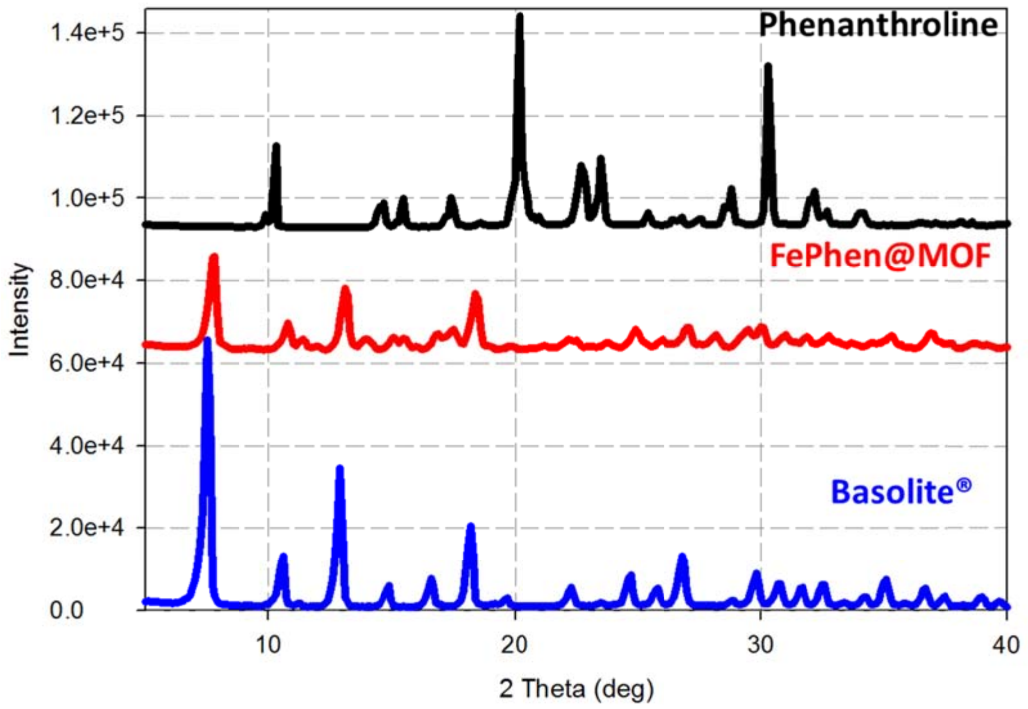

Supplementary Figure 1 | X-ray diffraction of catalyst precursors. Powder pattern comparison of non-heat treated Basolite Z1200®, FePhen@MOF and 1, 10-phenanthroline to confirm formation of MOF structure with encapsulation synthesis of FePhen@MOF and absence of crystalline 1,10-phenanthroline.

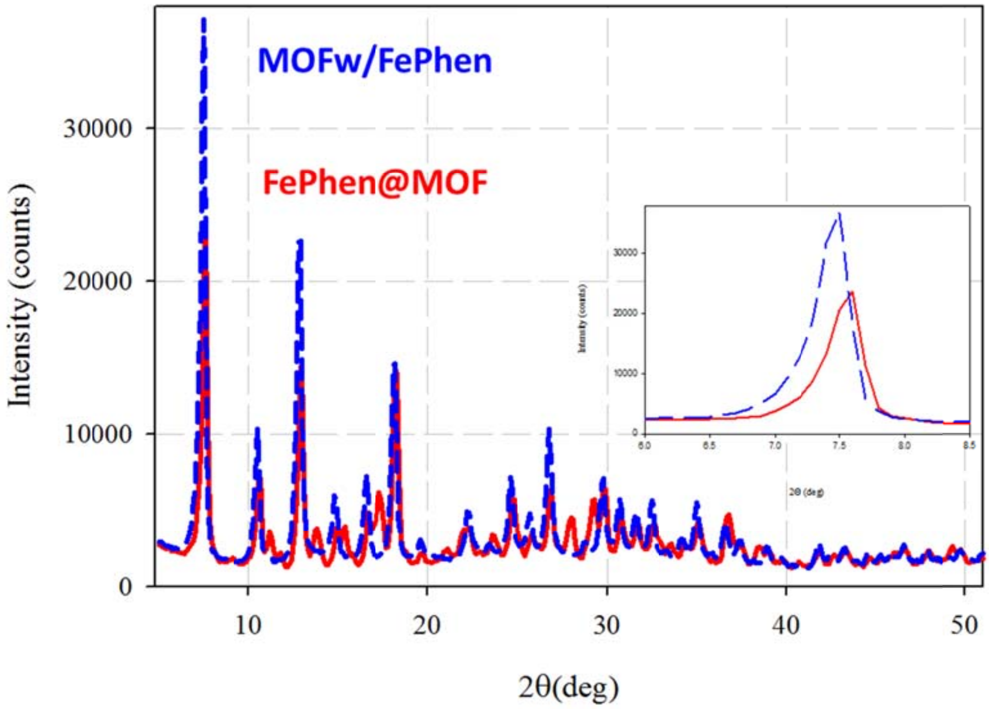

Supplementary Figure 2 | Effect of encapsulation on X-ray diffraction pattern. Powder pattern comparison of two samples: ZIF-8 MOF mixed with FePhen complex (MOFw/FePhen) and FePhen@MOF from encapsulation synthesis. First peak of FePhen@MOF compared to MOFw/FePhen (inset) shows diminished intensity and positive shift due to encapsulation.

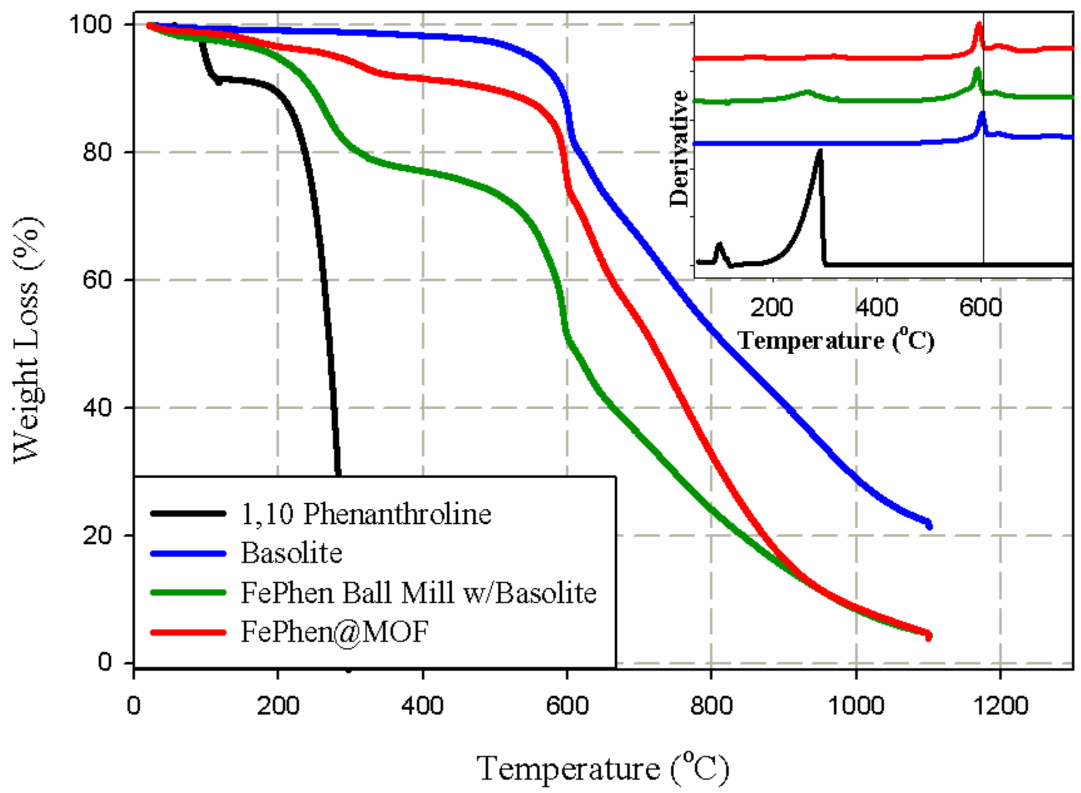

Supplementary Figure 3 | Thermogravimetric analysis of catalyst precursors. Thermogravimetric analysis of 1,10 Phenanthroline precursor, Basolite Z1200®, FePhen precurors mixed with Basolite Z1200® (MOFw/FePhen), and non-heat treated FePhen@MOF. Thermal stability of 1,10-phenanthroline in enhanced due to encapsulation in FePhen@MOF. All measurements were run under argon with 100 mL min^{-1} flow rate. Samples were heated at ramp rate of 5 °C min^{-1}.

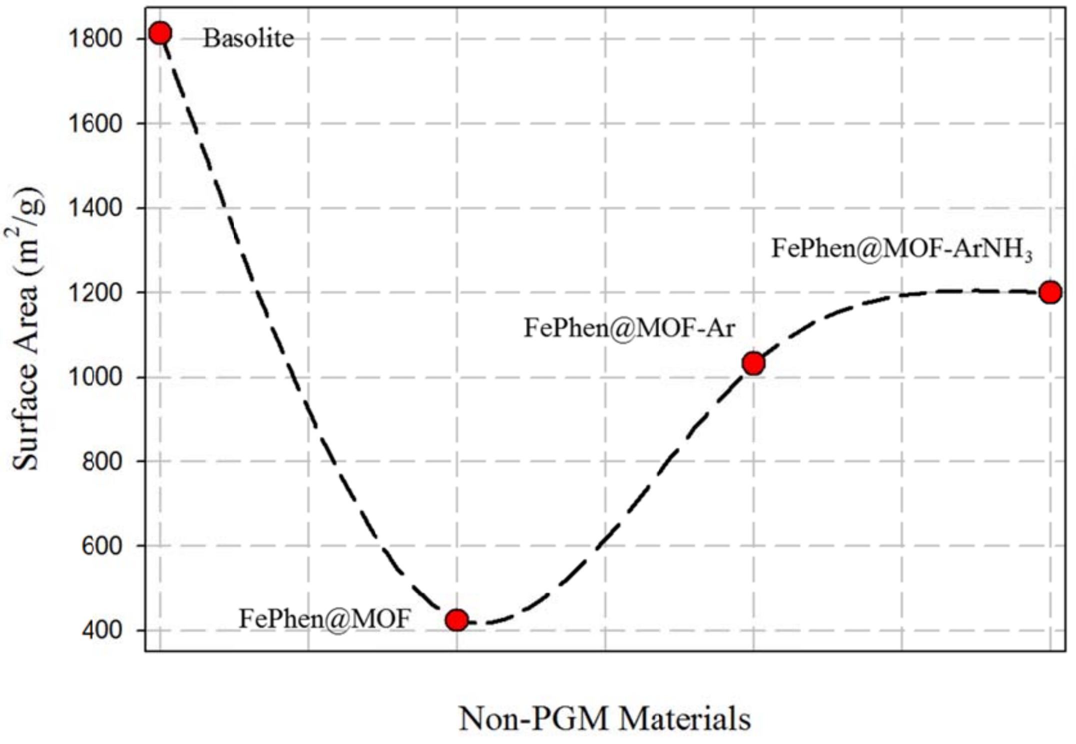

Supplementary Figure 4 | Surface area comparison. Brunauer-Emmett-Teller (BET) surface area of non-heat treated Basolite Z1200®, FePhen@MOF, FePhen@MOF-Ar and FePhen@MOF-ArNH$_3$. Encapsulation synthesis of FePhen@MOF results in diminished surface area, but a high surface area is recovered with heat treatments.

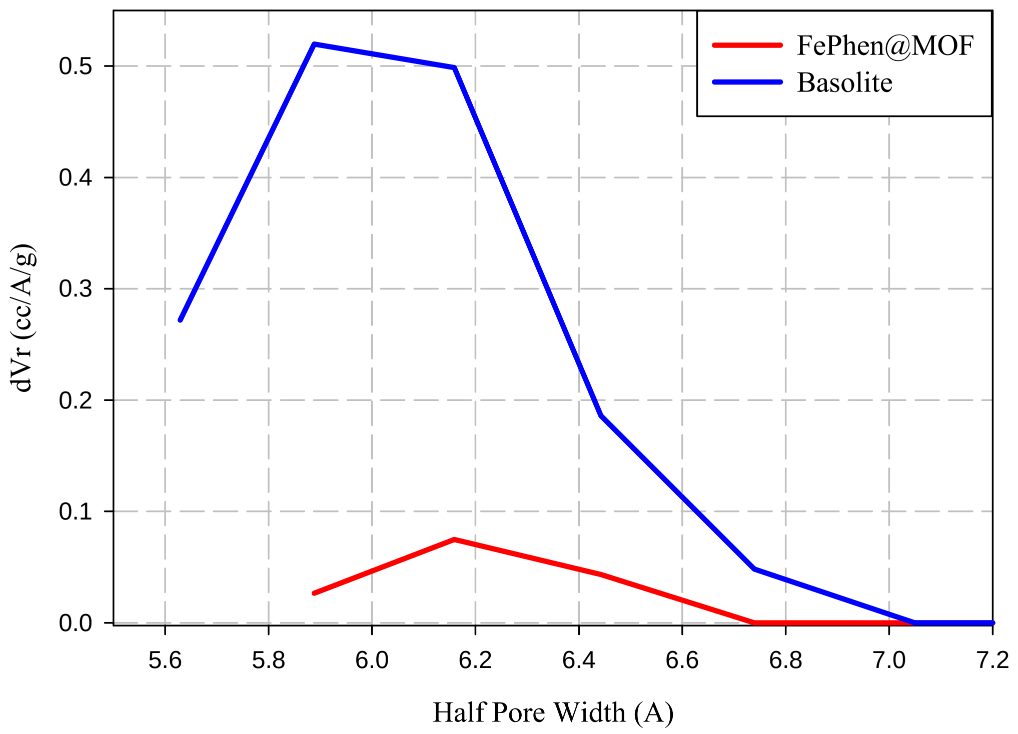

Supplementary Figure 5 | Change of pore size distribution from encapsulation. Pore size distribution of Basolite Z1200® and FePhen@MOF determined from Non-Local Density Functional Theory (NLDFT) model of nitrogen adsorption data. The pore volume of FePhen@MOF is less than Basolite Z1200® due to encapsulation of FePhen in the pores.

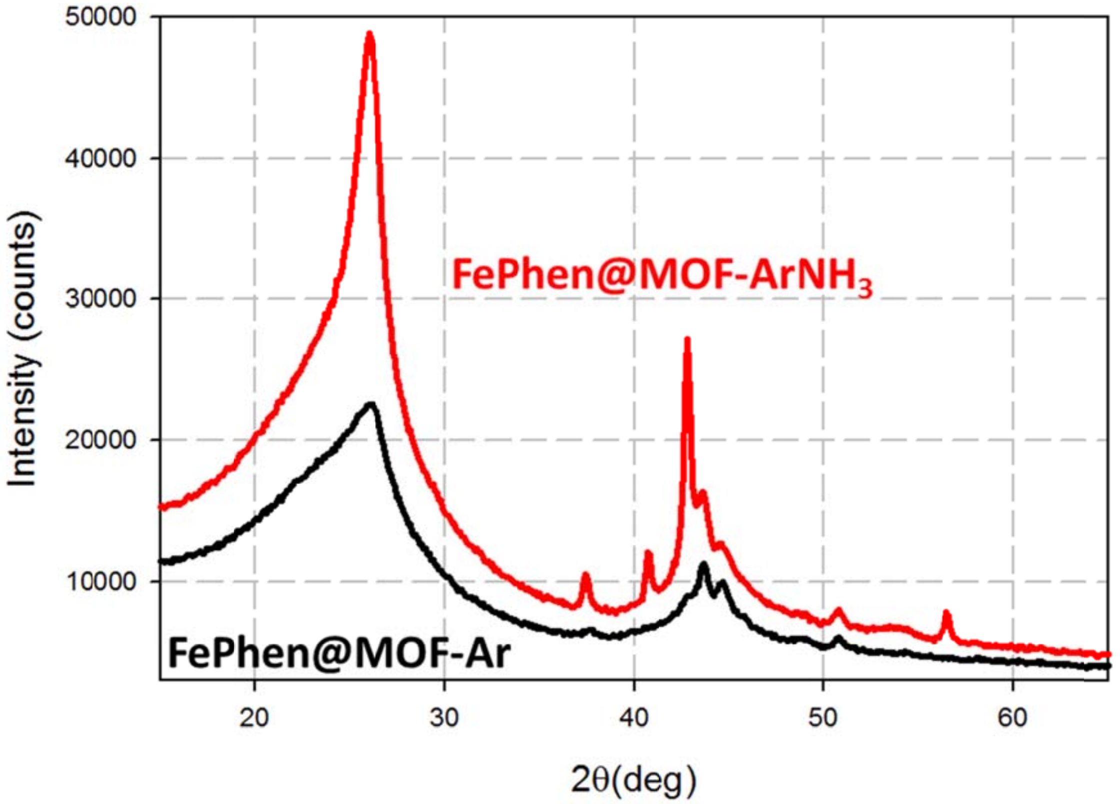

Supplementary Figure 6 | Incorporation of nitrogen after ammonia heat treatment. Powder pattern comparison of FePhen@MOF-Ar (heat treated in argon) and FePhen@MOF-ArNH_3 (heat treated in argon followed by ammonia), to show effect of ammonia heat treatment of FePhen@MOF. Subsequent heat treatment in ammonia shows emergence of iron nitride diffraction lines indicating incorporation of nitrogen functionalities.

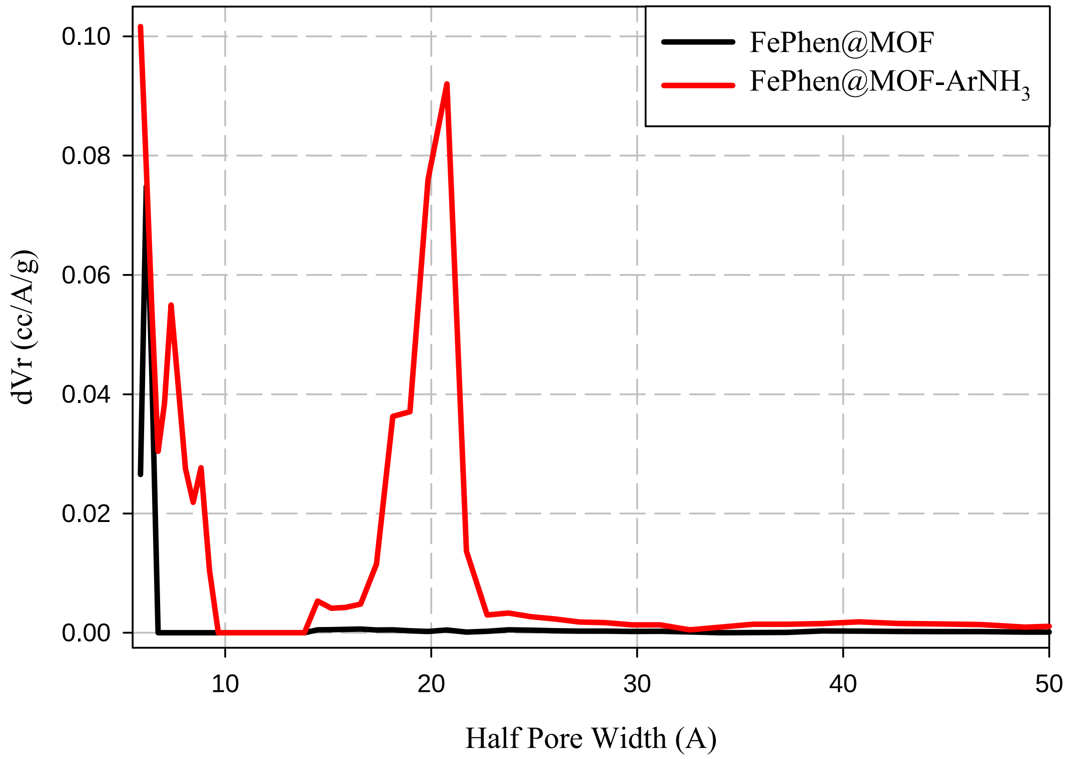

Supplementary Figure 7 | Change of pore size distribution from heat treatment. Pore size distribution of non-heat treated FePhen@MOF compared to FePhen@MOF-ArNH_3, determined from Non-Local Density Functional Theory (NLDFT) model of nitrogen adsorption data. Heat treatment introduces mesopores (20-40 Å) and new micropores (<20 Å).

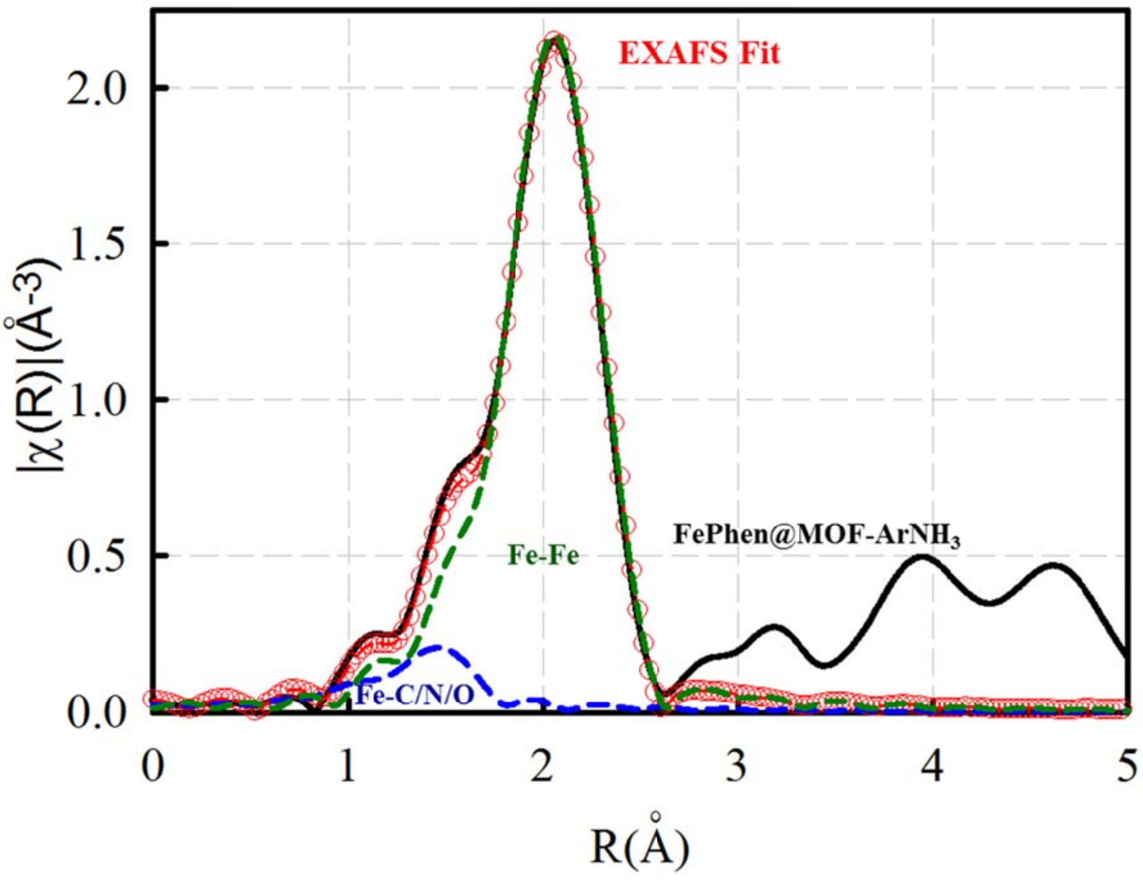

Supplementary Figure 8 | In situ EXAFS fit of FePhen@MOF-ArNH$_3$. Fe-K-edge non-phase corrected Fourier transformed EXAFS spectrum of FePhen@MOF-ArNH$_3$ collected under *in situ* conditions in N_2 saturated 0.1 M $HClO_4$ held at 0.3V vs. RHE. See Supplementary Table 3 for the corresponding EXAFS fit results.

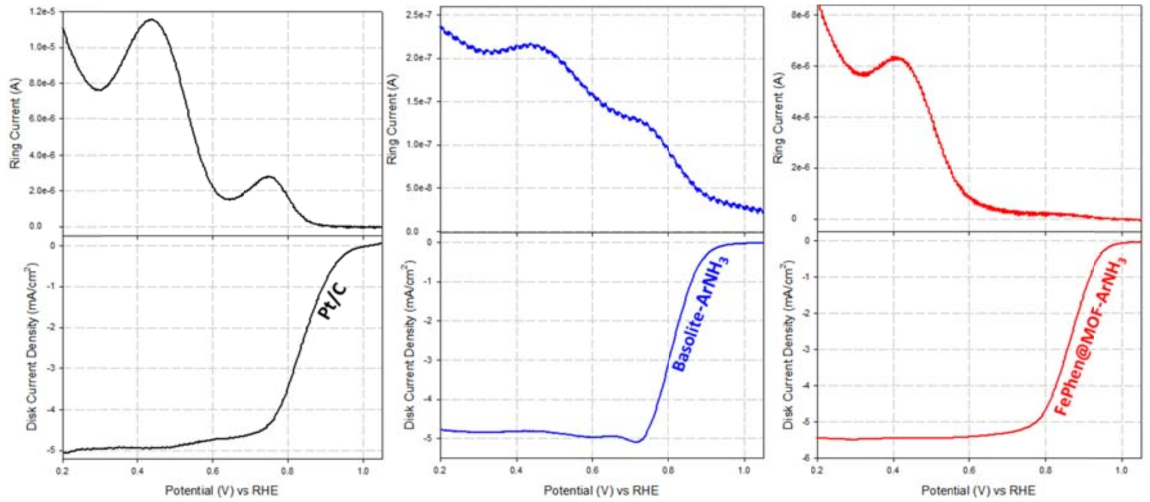

Supplementary Figure 9 | Stabilization of peroxide intermediate in alkaline media. Disk- and ring-currents measured during oxygen reduction reaction (ORR) on Tanaka Pt/C, Basolite-$ArNH_3$ and FePhen@MOF-$ArNH_3$ in O_2 saturated 0.1M KOH electrolyte at 20 mV s^{-1} with rotation rate of 1600 rpm at room temperature. E_{ring}=1.1 V vs RHE. Ring-current due to peroxide oxidation coincides with onset of reduction at the disk or Pt/C and Basolite-$ArNH_3$, however with FePhen@MOF-$ArNH_3$ oxidation of peroxide is shifted cathodically and is attributed to the 2-electron reduction of oxygen by the quinone functionalities on the carbon.

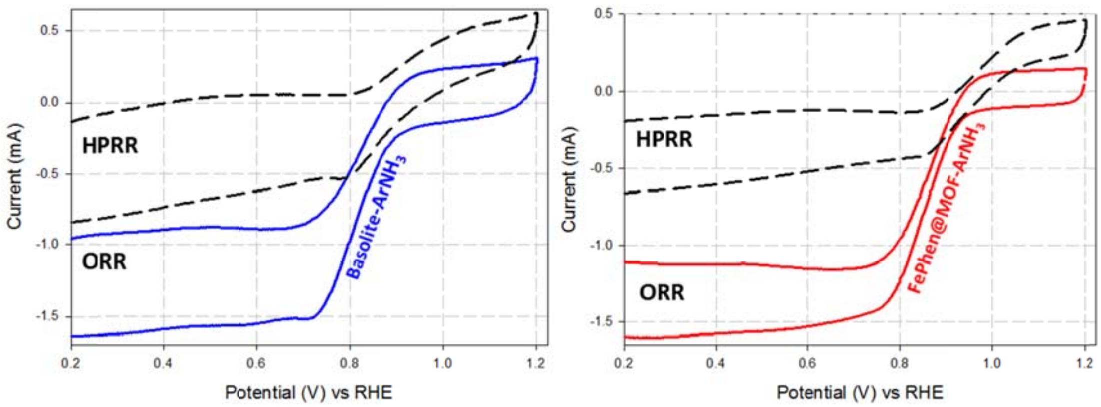

Supplementary Figure 10 | Activity for hydrogen peroxide reduction in alkaline media. Disk-currents measured on Basolite-ArNH$_3$ and FePhen@MOF-ArNH$_3$ in O_2 saturated 0.1M KOH electrolyte at 20 mV s^{-1} with rotation rate of 1600 rpm at room temperature. Hydrogen peroxide reduction reaction (HPRR) is shown in argon saturated electrolyte with 3.5 mM H_2O_2 added externally. Basolite-ArNH$_3$ and FePhen@MOF-ArNH$_3$ did not show HPRR activity.

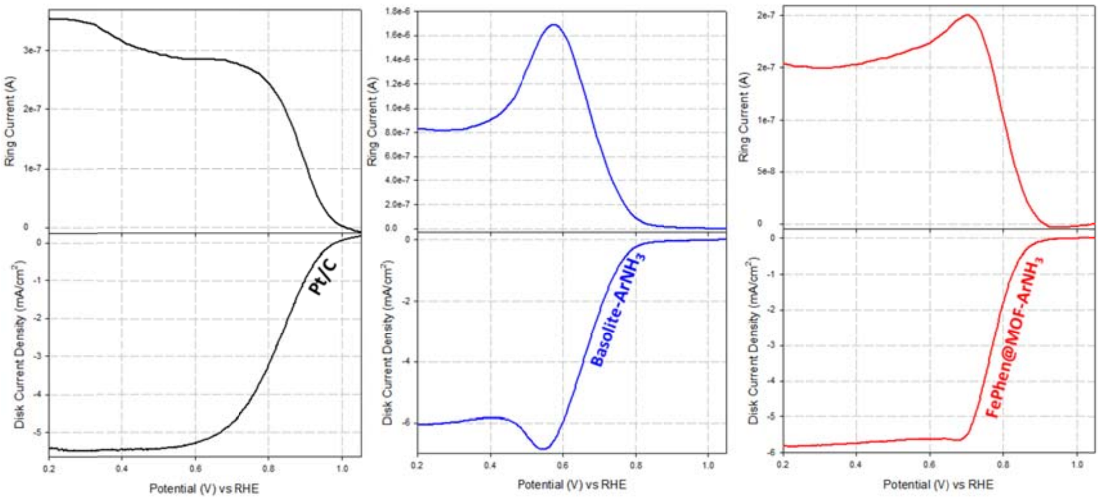

Supplementary Figure 11 | Stabilization of peroxide intermediate in acidic media. Disk- and ring-currents measured during oxygen reduction reaction (ORR) on Tanaka Pt/C, Basolite-$ArNH_3$, and FePhen@MOF-$ArNH_3$ in O_2 saturated 0.1M $HClO_4$ electrolyte at 20 mV s^{-1} with rotation rate of 1600 rpm at room temperature. E_{ring}=1.3 V vs RHE. Ring-current due to peroxide oxidation coincides with onset of oxygen reduction at the disk for all catalysts, indicating peroxide intermediate is not stable in acidic media. However, the magnitude of the ring-current for Basolite-$ArNH_3$ is an order of magnitude higher than FePhen@MOF-$ArNH_3$ indicating the presence of Fe promotes the selectivity for the 4e^- ORR pathway on FePhen@MOF-$ArNH_3$.

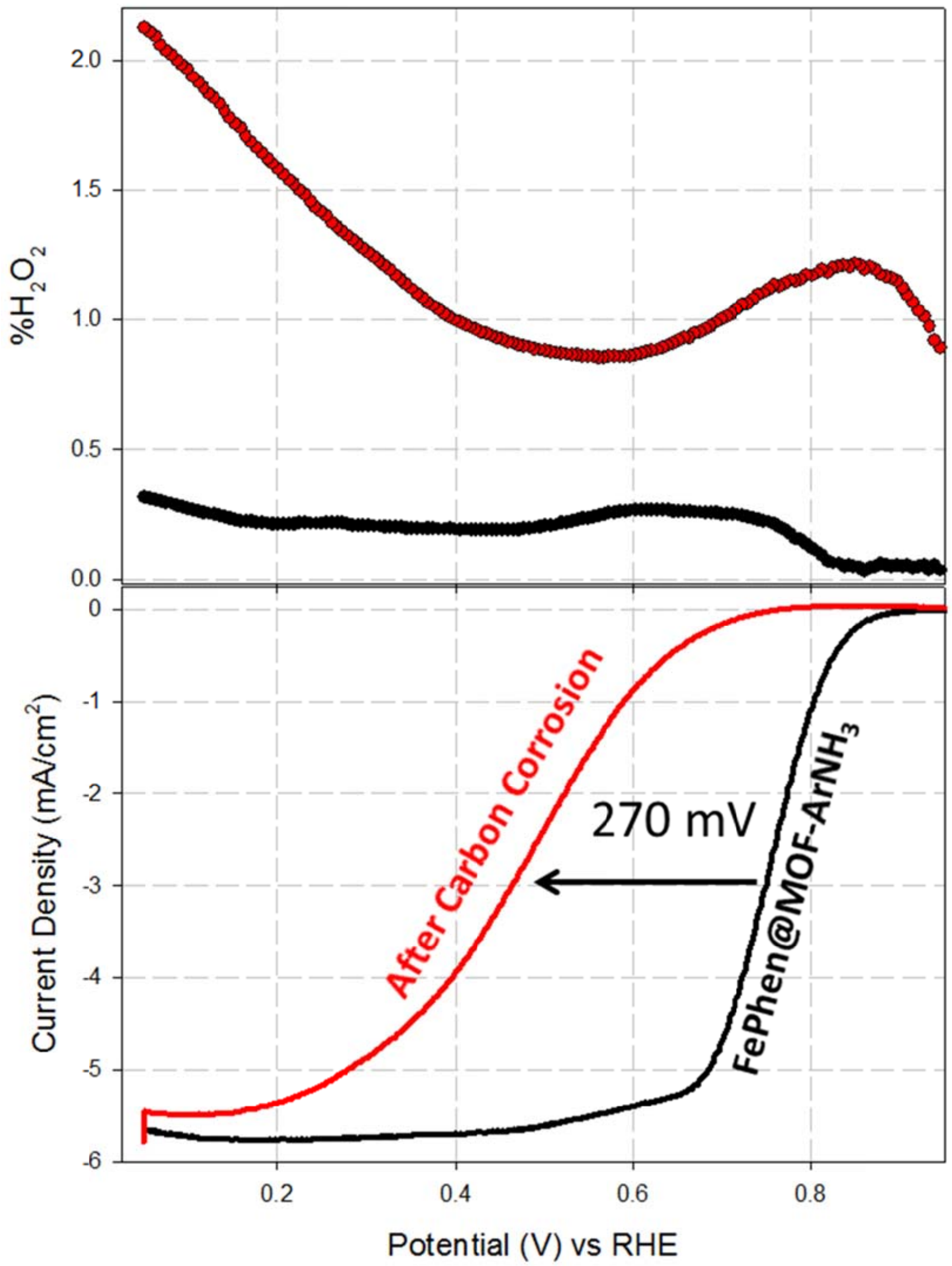

Supplementary Figure 12 | Effect of carbon corrosion on electrocatalytic activity. Disk- and ring-currents measured during oxygen reduction reaction (ORR) on FePhen@MOF-ArNH$_3$ in oxygen saturated 0.1M HClO$_4$ at 20 mV s^{-1} with a rotation rate of 1600 rpm at room temperature. E_{ring}=1.3 V vs RHE. FePhen@MOF-ArNH$_3$ ORR polarization plots initially and after 6,200 cycles (1.0 – 1.5 V vs RHE at 100 mV s^{-1} and rotation rate of 1600 rpm in argon saturated 0.1 M HClO$_4$).

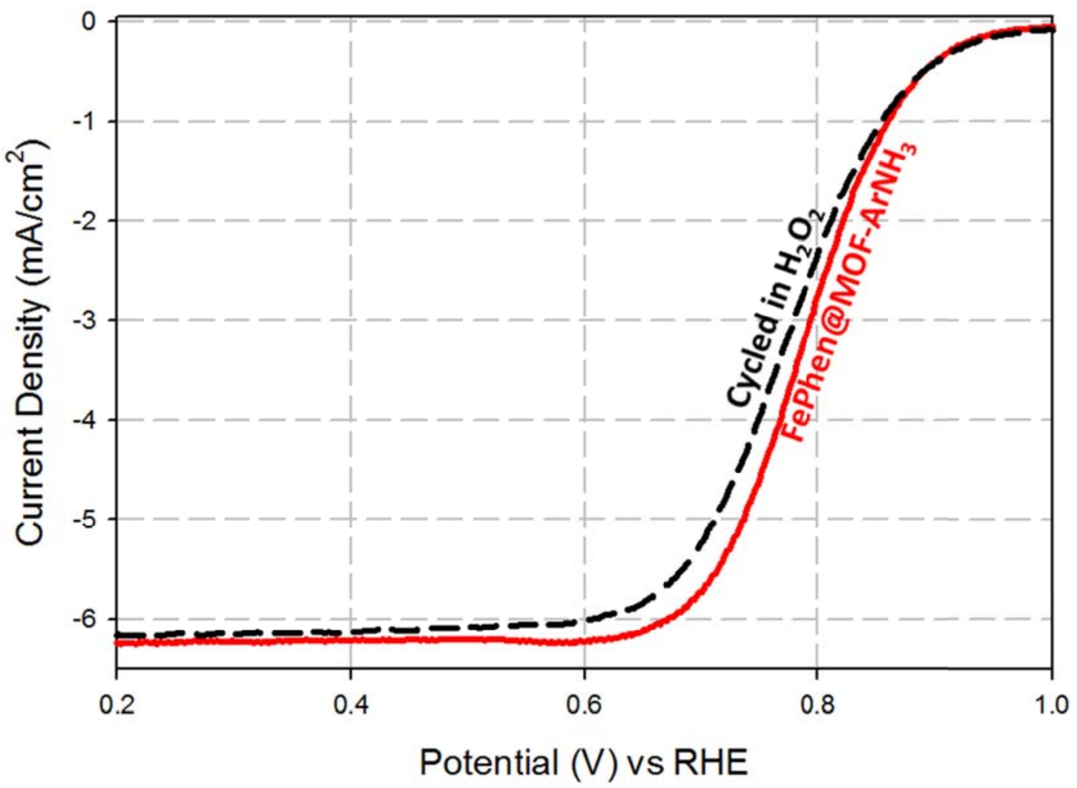

Supplementary Figure 13 | Effect of hydrogen peroxide cycling on electrocatalytic activity. Disk-currents measured during oxygen reduction reaction (ORR) on FePhen@MOF-ArNH$_3$ in oxygen saturated 0.5M H_2SO_4 at 20 mV s^{-1} with a rotation rate of 1600 rpm at room temperature. FePhen@MOF-ArNH$_3$ ORR polarization plots initially and after cycling (0.5 – 1.2 V vs RHE at 50 mV s^{-1} and rotation rate of 1600 rpm in argon saturated 0.5 M H_2SO_4 with 70mM H_2O_2). More detailed discussion in Supplementary Note 6.

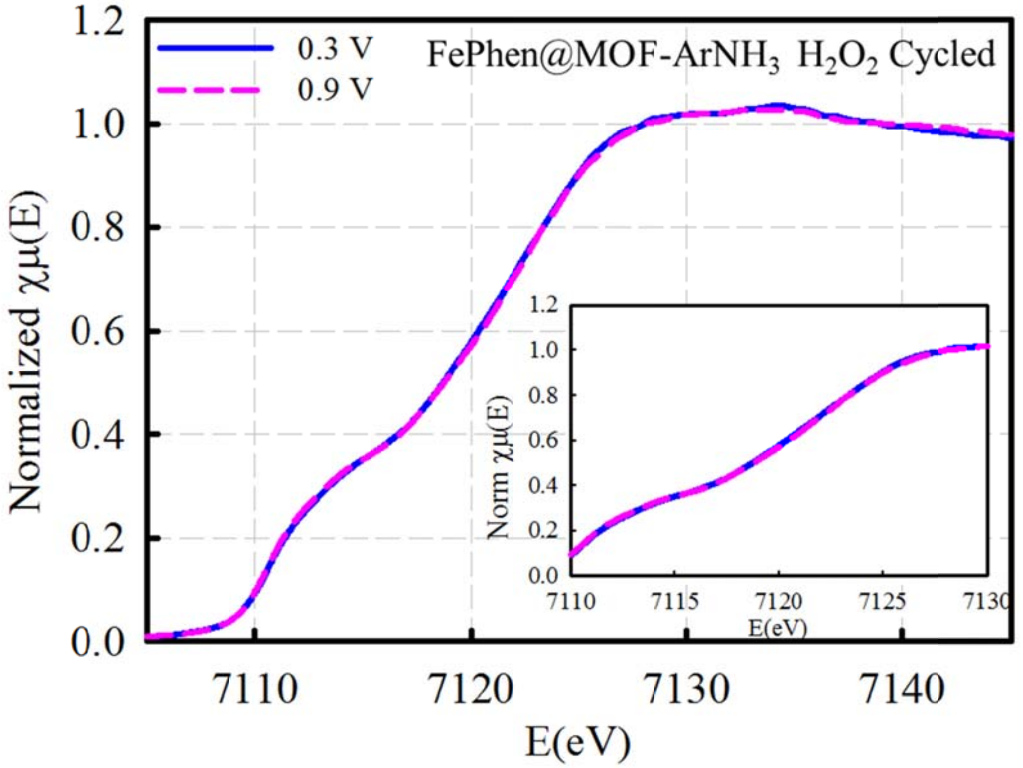

Supplementary Figure 14 | Effect of hydrogen peroxide cycling on XANES. Potential dependent normalized Fe K-edge XANES of H_2O_2 cycled (1.2-0.05 V vs RHE, 70mM H_2O_2 in 0.5 M H_2SO_4) FePhen@MOF-ArNH_3 collected in nitrogen saturated 0.1M $HClO_4$, at 0.3 and 0.9 V vs RHE.

Supplementary Tables

Sample	Imax (nm)	A	%wt phenanthroline
1,10-phenanthroline	287	0.114-1.467	n/A
Basolite Z1200©	203	0.044	0
FePhen@MOF	286	0.274	4.26

Supplementary Table 1 | Quantification of phenanthroline retained after encapsulation. Quantification of 1,10-phenanthroline in non-heat treated Basolite Z1200© and FePhen@MOF to confirm retention after encapsulation synthesis.

**EXAFS**	**FePhen@MOF-ArNH$_3$**	
**@ 0.3 V**	**Fe-C/N/O**	**Fe-Fe**
**CN**	1.2 ± 0.7	6.8 ± 1.2
**R (Å)**	1.97 ± 0.06	2.51 ± 0.01

Supplementary Table 2 | *In situ* EXAFS fit results. EXAFS fit results for FePhen@MOF-ArNH$_3$. Experiments performed at the Fe K-edge (7112 eV) as a function of potential in nitrogen saturated 0.1M $HClO_4$ electrolyte. Coordination number (CN) and phase-corrected bond length (R) in angstrom are shown.

	γ-Fe	α-Fe	Doublet	Fe_3C
IS[(1)] / mm s^{-1}	- 0.08[(2)]	- 0.03	0.16	0.19
QS / mm s^{-1}	0.00	0.00	0.50	0.00
LW / mm s^{-1}	0.29	0.40	0.54	0.42
HF / Tesla	0.00	33.2	0.00	20.7
% Area	13	6	45	36

Supplementary Table 3 | Table of fitted Mössbauer parameters. Fitted spectral parameters for the four components used to fit the ^{57}Fe Mössbauer spectrum of FePhen@MOF-ArNH_3. The isomer shift (IS), quadrupole splitting (QS), linewidth, (LW) and hyperfine field (HF) are given as well as the relative absorption area corresponding to each component (% Area).

[(1)] Relative to a calibration performed with an α-Fe foil.

[(2)] Fixed spectral parameter, on the basis of previous studies on Fe-N-C catalysts.

SAMPLE	%Fe	%Zn
FePhen@MOF	0.65	21.85
FePhen@MOF-Ar	2.5	0.56
FePhen@MOF-ArNH_3	3.1	0.13
Basolite	-	25

Supplementary Table 4 | Quantification of iron and zinc. Quantification of Fe and Zn in non-PGM materials based on inductively coupled plasma mass spectrometry (ICP-MS) measurements (0.012 gm/L limit of detection).

Supplementary Notes

Supplementary Note 1 | Evidence of encapsulation

It is well recognized that an unambiguous proof of encapsulation of FePhen within the MOF structure is very difficult. This is exemplified in a past report[1], however our conclusion arrived after careful analysis of a multitude of analytical tools (XRD, TGA, BET and UV-Vis analysis) provides the strongest evidence possible for such an encapsulation. Encapsulation of three phenanthrolines per Fe is not achieved during this encapsulation due to obvious steric considerations, but it is our contention is that opposed to earlier reports where such chelation could be the logical course, the unique environment enabled by encapsulation allows for preferential 1:1 chelation of Phen:Fe. This is the source of the unique evolution of the catalyst described in this report.

Best analytical proof for encapsulation is provided by comparison of XRD powder patterns of two samples i.e., the MOF mixed with FePhen complex (MOFw/FePhen, same molar ratio) and FePhen@MOF from the encapsulation synthesis (in which we speculate the Fe-complex is within the pores). As shown in Supplementary Figure 2, there is a slight positive shift in the principle powder pattern of FePhen@MOF, as compared to MOFw/FePhen. This shift could be attributed to the interactions between the walls of the MOF pores contributing secondary sphere interactions with the encapsulated iron-phenanthroline complex, consequently contracting the MOF pore walls more closely around the complex thus resulting in a reduced d-spacing and the consequent positive shift observed for FePhen@MOF. Similar shift could arguably be a result of some incorporation of Fe as nodes within the MOF structure. However this is highly unlikely since such a shift would only be possible with a significantly higher molar ration of Fe in the synthesis. Additionally, when chemical encapsulation is attempted with Fe

without chelation involving phenanthroline the desired MOF structure does not form. Chelation of the iron with the MOF ligand, 2-methylimidazole competes with 2-methylimidazole/zinc chelation therefore disrupting proper formation of the skeletal framework. 1,10-phenanthroline is necessary as a superior chelation agent for iron so that ZIF-8 can successfully manifest around the phenathroline/iron complex.

In addition, our thermogravimetric analysis (TGA) (Supplementary Fig. 3) determined that the weight loss below 300°C was due to decomposition of 1,10-phenanthroline and weight loss above 500°C was due to decomposition of ZIF-8 MOF structure. The thermal behavior of FePhen@MOF when compared with MOFw/FePhen indicates that for the former (Fe-Phen@MOF) there is a reduced mass loss of 1,10-phenanthroline. This clearly indicates the relative absence of free 1,10-phenanthroline in FePhen @MOF and the encapsulation engendered thermal stabilization of the phenanthroline. TGA results of FePhen@MOF (Supplementary Fig. 3) show the most significant mass loss occurring above 500 °C, due to the evaporation of zinc from the ZIF-8 structure, and above 600 °C the decomposition of the iron complex thus forming agglomerated metallic iron nanoparticles which catalyze the graphitization of the vaporized carbon. This explains partly the formation of carbo-nitrided fibers with encapsulated Fe carbides.

BET analysis shows a severe diminution of surface area in FePhen@MOF compared to Basolite (Supplementary Fig. 4), which we interpret as indicative of the iron and nitrogen precursors inhabiting the pores of the synthesized MOF. Additionally, the pore size distribution of FePhen@MOF is narrower than Basolite (Supplementary Fig. 5) which could be attributed to loss of the larger pores due to encapsulation of the iron and nitrogen precursors. We assume the

length of 1,10-phenanthroline, ~7 Å, is the limiting molecular size and is small enough to fit inside the MOF pore, 11.4 Å.

Confirmation of ZIF-8 formation, absence of 1,10-phenanthroline powder pattern, the positive shift of the powder pattern, thermal stabilization of 1,10-phenanthroline and diminished BET surface area in FePhen@MOF strongly support that the FePhen complex is not dispersed on the surface of the MOF, but rather encapsulated within the pores.

Supplementary Note 2 | X-ray Absorption Spectroscopy (XAS) Analysis

Electrode Preparation and XAS Data Collection. The electrode inks for the EXAFS electrodes were composed of 1:1 (wt%) 18.2 MΩ purity deionized water (Millipore) and 2-propanol (HPLC-grade, Aldrich), a 5 wt% Nafion solution (Aldrich), and the catalyst powder. The composition was chosen to give a final electrode with a dry Nafion loading of 5 wt%. The ink was hand-painted onto a Zoltek® carbon cloth and dried for 15 minutes in a 65°C vacuum oven between coats. The final iron geometric loadings were chosen to give 0.05 edge heights at the Fe K–edge. The spectro−electrochemical cell was flooded with N_2 or O_2-saturated 0.1 M $HClO_4$ electrolyte during operation. Full range Fe K–edge spectra were taken at various static potentials along the anodic sweep of the Cyclic voltammogram (CV). Data were collected in fluorescence mode with a Fe reference foil positioned between I_2 and I_3 as a reference. The voltage cycling limits were 0.05 to 1.10 V vs. RHE. Data collection was performed at the chosen potentials held during anodic sweeps. Before each measurement, the cell was held for 5 minutes to reach a pseudo-steady state. The electrode was fully cycled following each potential hold in order to clean the electrocatalyst surfaces after each potential hold.

Scans were calibrated, aligned and normalized with background removed using the IFEFFIT suite.[2] The data was processed and fitted using the Athena[2] and Artemis[3] programs. The $\chi(R)$ transforms were modeled using scattering paths calculated by the FEFF6 code.[4] Data analysis for Delta−Mu ($\Delta\mu$) studies at the Fe K−edge involved specific normalization procedures detailed elsewhere.[5] Difference spectra were obtained using the equation

$$\Delta\mu = \mu(V, \text{Ar or } O_2) - \mu(0.3\ V, \text{Ar}) \qquad (1)$$

where $\mu(V, \text{Ar or } O_2)$ is the XANES of the catalyst at various potentials in Ar− or O_2−saturated electrolyte, and $\mu(0.1\ V, \text{Ar})$ is the reference XANES signal at 0.1 V in Ar−saturated electrolyte

at which potential no evidence for electrochemical adsorbates (H*, O(H)*) were found on these Fe−based catalysts.

EXAFS Fits. Traditional Fourier Transform (FT) EXAFS analysis up to the second shell (for convenience, the shell associated with the first and second FT peak is named as the first and second shell, respectively) is applied at the Fe K−edge for representative data at 0.3 V for FePhen@MOF-ArNH_3 (Supplementary Fig. 8). The fitting results are summarized in Supplementary Table 2.

Supplementary Note 3 | Discussion of detection of FeN$_x$ species using *in situ* XAS:

Visual examination of the EXAFS data cannot rule out the presence of small amount of Fe-N$_x$ species by itself, especially considering the normal uncertainties of EXAFS fits (usually <1 for coordination number). In this work EXAFS is only used as additional evidence of the absence of Fe-N$_x$ species. This conclusion is mainly drawn from the XANES side (Figure 3c), which is much more sensitive than EXAFS. More importantly, *in situ* XANES is extremely powerful to capture the key aspect of the Fe-N$_x$ species during voltage cycling: the redox $Fe^{2+/3+}$ transition ($N_x{-}Fe^{II} + H_2O \rightarrow N_x{-}Fe^{III}{-}OH_{ads} + H^+ + e^-$). As shown in Figure 3c top right, as for the Fe-based catalysts containing the Fe-N$_x$ species, the Fe K−edge shifts toward higher energy indicating the Fe^{2+}/Fe^{3+} redox transition (this is also why we present the XAS data of these two representative catalysts). This shift arises exclusively from the Fe-N$_x$ species since the valence state of the metallic Fe encapsulated in carbon matrix does not change with applied potential. On the other hand, no XANES shift was observed on the target catalyst. Note the shift magnitude decreases with the decreasing relative content of Fe-N$_x$ species owing to the bulk averageing nature of XAS. However, the Fe K−edge shift magnitude for one Fe-N$_x$ undergoing $Fe^{2+/3+}$ transition is > 2.5 eV (Figure 3c).[6] Thus as long as the relative content of Fe-N$_x$ species is higher than 2% (which means 2% Fe-N$_x$ species and 98% metallic Fe in terms of atomic ratio of Fe), the shift (> 0.05 eV) will be readily captured by XANES or more sensitive Δμ-XANES. Thus even though there is some Fe-N$_x$ species that is beyond the sensitivity of the XANES, the content must be extremely low, which apparently cannot account for the exceptional ORR activity of this catalyst.

Supplementary Note 4 | Mossbauer spectroscopy analysis.

The singlet with an isomer shift (IS) of - 0.08 mm s^{-1} is ubiquitous in pyrolyzed Fe-N-C catalysts and assigned to paramagnetic γ-Fe[7-10]. While α-Fe nanoparticles could also result at RT in a singlet with similar parameters, it would turn into a sextet at 5 K due to a super-paramagnetic transition. The Mössbauer spectrum of FePhen@MOF-ArNH_3 measured at 5 K (not shown) still showed the singlet, thereby foreboding its assignment to α-Fe nanoparticles. Next, the most intense sextet in Figure 1g with a IS of 0.19 mm s^{-1} and a hyperfine field (HF) of 20.7 T corresponds to ferromagnetic Fe_3C[10]. The minor sextet with an IS-value close to zero and a larger hyperfine field of 33.2 T is unambiguously assigned to ferromagnetic α-Fe. The last component is a doublet with an IS of 0.16 mm s^{-1} and a quadrupole splitting (QS) of 0.50 mm s^{-1}. It may not be assigned to iron-rich nitrides such as ε-Fe_3N and γ'-Fe_4N (see supplementary information) but can be assigned to either paramagnetic amorphous iron carbide, Fe_xC[11,12] or nitrogen-rich iron nitrides[13]. On the basis of the experimental X-ray diffraction pattern (Fig. 1a) and doublet's Mössbauer parameters (especially QS), the doublet is assigned to ξ-Fe_2N or ε-$Fe_{2+x}N$ with x = 0.1, featuring an interstitial binary nitride with Fe atoms defining a hexagonal close-packed structure[13]. Its presence after heat treatment at 1050°C in pure NH_3 is in agreement with the Fe-N phase diagram and actually expected since the pyrolysis conditions are typically those employed for producing Fe_2N[14,15].

Thus, the analysis of the Mössbauer spectrum of FePhen@MOF-ArNH_3 demonstrates the absence of the doublets D1 and D2 that had been identified in all Fe-based non-PGM catalysts investigated with Mössbauer spectroscopy thus far. D1 and D2 have been assigned to FeN_xC_y moieties covalently integrated in graphene sheets, with the ferrous ion in low- and medium-spin state, respectively[16,17]. Their Mössbauer spectral parameters resemble those of Fe(II)-

phthalocyanine adsorbed on carbon (D1) and crystalline iron phthalocyanine (D2). The IS and QS values of D1 typically range from 0.26-0.39 mm s^{-1} and 0.90-1.16 mm s^{-1}, respectively[8,18]. For D2, the typical range of IS and QS values are 0.36-0.37 mm s^{-1} and 2.40-2.65 mm s^{-1}, respectively.

Supplementary Note 5 | Discussion on the assignment of the doublet in Mössbauer spectroscopy:

Iron-rich nitrides such as ε-Fe_3N and γ'-Fe_4N are usually ferromagnetic at RT and result in sextets in Mössbauer spectra[19]. As for α-Fe, nanoparticles of ε-Fe_3N and γ'-Fe_4N may however be paramagnetic at RT, leading to a doublet in a Mössbauer spectrum. They however become ferromagnetic again at low temperature, resulting in a sextet[14]. The present doublet, observed at RT and at 5 K (the latter spectrum is not shown), can therefore not be assigned to iron-rich nitrides.

Supplementary Note 6 | Durability test with hydrogen peroxide cycling.

The hydrogen peroxide cycling test was exclusively directed towards addressing any issues related to (a) unstable exposed Fe and (b) stability of the carbon nitride structure, both being very sensitive to the presence of relatively high concentration of peroxide. FePhen@MOF-ArNH_3 showed remarkable stability (Supplementary Figure 13) under these harsh conditions, with no increase in overpotential and only a 10mV cathodic shift in half-wave potential ($E_{½}$). Our *in situ* XAS study supported our RDE results, revealing Fe was stable in the harsh environment and no FeN_x moieties were detected (Supplementary Figure 14). XANES is very sensitive technique for detecting the presence of FeN_x species and our *in situ* collection of spectra as a function of potential further enhances the sensitivity of this technique. As the potential is increased the Fe K-edge XANES remains unchanged, confirming the Fe in the peroxide cycled FePhen@MOF-ArNH_3 is still present as subsurface Fe/Fe_xC particles.

References

1 Lee, J. *et al.* Metal–organic framework materials as catalysts. *Chemical Society Reviews* **38**, 1450-1459 (2009).

2 Newville, M. IFEFFIT: Interactive XAFS Analysis and FEFF Fitting. *J. Synchrotron. Radiat.* **8**, 322-324 (2001).

3 Ravel, B. & Gallagher, K. Atomic structure and the magnetic properties of Zr-doped Sm2Co17. *Phys. Scr.* **T115**, 606-608 (2005).

4 Zabinsky, S. I., Rehr, J. J., Ankudinov, A., Albers, R. C. & Eller, M. J. Multiple-scattering calculations of x-ray-absorption spectra. *Physical Review B* **52**, 2995-3009 (1995).

5 Roth, C. *et al.* Determination of O[H] and CO Coverage and Adsorption Sites on PtRu Electrodes in an Operating PEM Fuel Cell. *Journal of the American Chemical Society* **127**, 14607-14615, doi:10.1021/ja050139f (2005).

6 Bae, I. T., Tryk, D. A. & Scherson, D. A. Effect of Heat Treatment on the Redox Properties of Iron Porphyrins Adsorbed on High Area Carbon in Acid Electrolytes: An in Situ Fe K-Edge X-ray Absorption Near-Edge Structure Study. *Journal of Physical Chemistry B* **102**, 4114-4117, doi:10.1021/jp972605w (1998).

7 Goellner, V. *et al.* Degradation of Fe/N/C catalysts upon high polarization in acid medium. *Physical Chemistry Chemical Physics* **16**, 18454-18462 (2014).

8 Kramm, U. I., Lefèvre, M., Larouche, N., Schmeisser, D. & Dodelet, J.-P. Correlations between Mass Activity and Physicochemical Properties of Fe/N/C Catalysts for the ORR in PEM Fuel Cell via 57Fe Mössbauer Spectroscopy and Other Techniques. *Journal of the American Chemical Society* **136**, 978-985 (2014).

9 Morozan, A. *et al.* Effect of Furfuryl Alcohol on Metal Organic Framework-based Fe/N/C Electrocatalysts for Polymer Electrolyte Membrane Fuel Cells. *Electrochimica Acta* **119**, 192-205 (2014).

10 Ruskov, T. *et al.* Mössbauer transmission and back scattered conversion electron study of Fe nanowires encapsulated in multiwalled carbon nanotubes. *Journal of applied physics* **96**, 7514-7518 (2004).

11 Miyatani, R., Yamada, Y. & Kobayashi, Y. Mössbauer study of iron carbide nanoparticles produced by sonochemical synthesis. *Journal of Radioanalytical and Nuclear Chemistry*, 1-4 (2014).

12 Yamada, Y., Yoshida, H., Kouno, K. & Kobayashi, Y. in *Journal of Physics: Conference Series.* 012096 (IOP Publishing).

13 Borsa, D. & Boerma, D. Phase identification of iron nitrides and iron oxy-nitrides with Mössbauer spectroscopy. *Hyperfine interactions* **151**, 31-48 (2003).

14 Kurian, S. & Gajbhiye, N. Magnetic and Mössbauer study of ε-Fe y N (2< y< 3) nanoparticles. *Journal of Nanoparticle Research* **12**, 1197-1209 (2010).

15 Wang, L. *et al.* Ion-exchanged route synthesis of Fe 2 N–N-doped graphitic nanocarbons composite as advanced oxygen reduction electrocatalyst. *Chemical Communications* **49**, 3022-3024 (2013).

16 Koslowski, U. I., Abs-Wurmbach, I., Fiechter, S. & Bogdanoff, P. Nature of the catalytic centers of porphyrin-based electrocatalysts for the ORR: a correlation of kinetic current density with the site density of Fe− N4 centers. *The Journal of Physical Chemistry C* **112**, 15356-15366 (2008).

17 Kramm, U. I. *et al.* Structure of the catalytic sites in Fe/N/C-catalysts for O2-reduction in PEM fuel cells. *Physical Chemistry Chemical Physics* **14**, 11673-11688 (2012).
18 Jaouen, F. Heat-Treated Transition Metal-NxCy Electrocatalysts for the O2 Reduction Reaction in Acid PEM Fuel Cells. *Non-Noble Metal Fuel Cell Catalysts*, 29-118 (2013).
19 Kurian, S. & Gajbhiye, N. Low temperature and in-field Mössbauer spectroscopic studies of ε-Fe< sub> 3</sub> N particles synthesized from iron–citrate complex. *Chemical Physics Letters* **493**, 299-303 (2010).
